# Platelet-Derived Microvesicles Promote VSMC Dedifferentiation After Intimal Injury via Src/Lamtor1/mTORC1 Signaling

**DOI:** 10.3389/fcell.2021.744320

**Published:** 2021-09-16

**Authors:** Ji-Ting Liu, Han Bao, Yang-Jing Fan, Zi-Tong Li, Qing-Ping Yao, Yue Han, Ming-Liang Zhang, Zong-Lai Jiang, Ying-Xin Qi

**Affiliations:** ^1^Institute of Mechanobiology and Medical Engineering, School of Life Sciences and Biotechnology, Shanghai Jiao Tong University, Shanghai, China; ^2^Department of Endocrinology and Metabolism, Shanghai Jiao Tong University Affiliated Sixth People’s Hospital, Shanghai Diabetes Institute, Shanghai Clinical Center for Diabetes, Shanghai Key Laboratory of Diabetes Mellitus, Shanghai Key Clinical Center for Metabolic Disease, Shanghai, China

**Keywords:** intimal hyperplasia, platelet-derived microvesicles, vascular smooth muscle cells, dedifferentiation, Lamtor1

## Abstract

Phenotypic switch of vascular smooth muscle cells (VSMCs) is important in vascular remodeling which causes hyperplasia and restenosis after intimal injury. Platelets are activated at injured intima and secrete platelet-derived microvesicles (PMVs). Herein, we demonstrated the role of PMVs in VSMC phenotypic switch and the potential underlying mechanisms. *In vivo*, platelets were locally adhered and activated at intimal injury site, while Lamtor1 was promoted and VSMCs were dedifferentiated. PMVs, collected from collagen-activated platelets *in vitro* which mimicked collagen exposure during intimal injury, promoted VSMC dedifferentiation, induced Lamtor1 expression, and activated mTORC1 signaling, reflected by the phosphorylation of two downstream targets, i.e., S6K and 4E-BP1. Knockdown of Lamtor1 with small interfering RNA attenuated these processes induced by PMVs. Based on the previously published proteomic data, Ingenuity Pathway Analysis revealed that Src may participate in regulating effects of PMVs. Src inhibitor significantly reversed the effects of PMVs on VSMC dedifferentiation, Lamtor1 expression and mTORC1 activation. Furthermore, in SMC-specific *Lamtor1* knockout mice, intimal hyperplasia was markedly attenuated after intimal injury compared with the wild type. Our data suggested that PMVs secreted by activated platelets promoted VSMC dedifferentiation *via* Src/Lamtor1/mTORC1 signaling pathway. Lamtor1 may be a potential therapeutic target for intimal hyperplasia after injury.

## Introduction

Vascular endothelium, which plays crucial roles in maintaining vascular homeostasis and modulating vasomotor tone, is a monolayer of cells that lines the inner luminal surface of vascular tubes and provides a barrier between blood and tissues. Endothelial injury occurs in a number of vascular pathologies, including atherosclerosis, coronary artery bypass grafting surgery, percutaneous coronary intervention, angioplasty or stenting (Wu et al., 2017). The denudation of endothelial cell during injury exposes the underlying collagen, leading to a rapid deposition and activation of platelets, and promotes pathological proliferation and migration of vascular smooth muscle cells (VSMCs) to form neointimal hyperplasia, which ultimately leads to restenosis (Wu et al., 2017). The phenotypic switching of VSMCs is a fundamental step for proliferation and migration. VSMCs are physiologically in contractile phenotype and can dedifferentiate to synthetic state under various pathological conditions, which are characterized as repressed expression of contractile proteins including α-smooth muscle actin (SMA), SM-22α, calponin and SM myosin heavy chains (Sinha et al., 2014).

Numerous groups have reported that shortly after neointimal injury, platelets, activated by subendothelial collagen and characterized with expressed CD62P (also known as P-Selectin), release not only platelet-derived growth factor but also platelet-derived microvesicles (PMVs). Microvesicles (MVs), a kind of extracellular vesicles (EVs), are typically around 100–1000 nm in size (Akers et al., 2013). PMVs, the most abundant MVs in the circulation, are important regulators during hemostasis (Webber and Johnson, 1970), inflammation (Boilard et al., 2010), and angiogenesis (Lukasik et al., 2013).

Elevated levels of PMVs are observed in various cardiovascular diseases (Suades et al., 2015). Previous studies have demonstrated that the increased numbers of PMV is correlated with the thickness of carotid artery intima and the lipid-rich atherosclerotic plaques (Csongrádi et al., 2011). PMVs can facilitate leukocyte accumulation at the site of endothelial injury and enhance leukocyte infiltration to the intima *via* P-Selectin expressed on PMVs (Forlow et al., 2000). Interestingly, PMVs have been proved to trigger VSMC proliferation and migration from the media to the intima thereby enhancing intimal hyperplasia (Weber et al., 2000). However, the molecular mechanisms underlying this process remains largely unclear.

The late endosomal/lysosomal adaptor MAPK and mTOR activator 1 (Lamtor1), also named as p18, is a highly conserved lysosome-anchored protein for activation of mTOR complex 1 (mTORC1) (Bar-Peled et al., 2012). Lamtor1 plays a pivotal role in cell growth, membrane protein transport, and lysosome biogenesis. Previous studies showed that Lamtor1 deficiency results in enhancing of autophagy flux and apoptosis (Nowosad et al., 2020), and disrupting the osteoblast differentiation *via* reduction of YAP nuclear localization (Block et al., 2020). Moreover, ablation of Lamtor1 is fatal in the embryo (Filipek et al., 2017). Although numerous studies revealed the importance of Lamtor1 in cell homeostasis and dysfunctions, the role of Lamtor1 in intimal hyperplasia caused by injury, and whether Lamtor1 is regulated by PMVs are still unknown.

In our present study, using intimal injury model and smooth muscle cell-specific (SMC-specific) *Lamtor1* knockout (KO) mice, the effects of Lamtor1 in VSMC differentiation and intimal injury were investigated, and then the potential roles of PMVs in this process were further detected.

## Materials and Methods

### SMC-Specific *Lamtor1*-KO Mice

To generation of SMC-specific *Lamtor1* KO mice with a C57BL/6J background, *Lamtor1*^fl/fl^ mice were crossed with α*-SM22*α*-Cre*^+^ mice which purchased from the Shanghai Model Organisms Center. The generated *Lamtor1*^fl/fl^*SM22*α*Cre*^+^ mice were genotyped by PCR, and littermate mice were used as controls. The SMC-specific *Lamtor1* KO mice and C57BL/6J background littermate controls were maintained on a light/dark (12/12 h) cycle at 25°C, and received food and water *ad libitum*.

The animal care and experimental protocols were conducted in accordance with the Animal Management Rules of China (Documentation 55, 2001, Ministry of Health, China), and the animal study was approved by the Animal Research Committee of Shanghai Jiao Tong University.

### Wire Injury Mouse Model

The carotid wire injury mouse model was constructed to mimic post-angioplasty restenosis in human (Berk et al., 1989). The left common carotid artery of each mouse was used as the experimental group, and the right one was the autologous control. In briefly, 10- to 12-week male C57BL/6J, SMC-specific *Lamtor1* KO mice or littermate control mice were anesthetized by 2% isoflurane at 1 L/min oxygen flow using an isoflurane vaporizer (Matrx VIP 3000). The left carotid artery was separated and the bifurcation area was exposed. The occipital artery, the internal carotid artery, and the external carotid artery were sequentially ligated using a surgical suture. Then a 0.3-mm diameter guide-wire (Advanced Cardiovascular Systems) was used to establish vascular intimal injury at the common carotid artery *via* the transverse arteriotomy of the external carotid artery.

### Van Gieson and Immunofluorescence Staining

Tissues were fixed in 4% paraformaldehyde, dehydrated in 30% sucrose and crossed into 6 μm sections (LEICA, RM2265). For Elastin Van-Gieson staining, sections were stained with Weigert Solution for 5 min, directly immerged into Differentiation Solution (1% hydrochloric acid alcohol), and then flushed with water. Van-Gieson Dye Solution was used to re-stain the sections for 5–6 min. A microscope (Olympus IX71) was used to observe images.

For immunofluorescent staining, sections or cells were fixed with 4% paraformaldehyde for 15 min, permeabilizated with 0.2% Triton X-100 for 3 min, and incubated with blocking buffer containing 10% goat serum for 1 h at room temperature. Antibody against CD62P (Biolegend, 1:100), Lamtor1 (CST, 1:200), phosphor-p70S6K (Thr389) (CST, 1:200), or p-Src (CST, 1:200) was diluted in blocking buffer and incubated overnight at 4°C. Sections or coverslips were washed with TBS three times and incubated with secondary antibody (Alexa Fluor 568-conjugated goat anti-rabbit IgG, 1:1000, Invitrogen) and α-SMA-FITC antibody (Sigma, 1:500) for 2 h at room temperature. Nuclei were stained with 4, 6-diamidino-2-phenylindole (DAPI) for 10 min. The images were obtained using a confocal laser scanning microscope (Fluoview 1000, Olympus).

### Carotid VSMCs Culture and Treatment

VSMCs were cultured from the carotid artery of male Sprague-Dawley (SD) rats (150–180 g) via an explant method (Berk et al., 1989). Briefly, the carotid artery was cut into small pieces and cultured in Dulbeco’s Modified Eagle medium (DMEM, Gibco) with 10% fetal bovine serum (FBS, Gibco), in a humidified incubator at 37°C, 5% CO_2_. Upon enrichment in 80–90% confluence, cells were trypsinized and seeded at required density for further assays.

For PMV treatment, cells were serum-starved for 24 h and then treated with PMVs (10^9^/mL) for 1 or 24 h at 37°C, 5% CO_2_. For Src inhibitor experiment, VSMCs were pre-incubated with Src inhibitor (Sigma, 10 μM) for 1 h and then treated with PMVs (10^9^/mL) for 24 h.

### Platelet Activation and PMVs Purification

Whole blood from abdominal aorta of anesthetized SD rats was collected into syringes containing 100 μL/mL anticoagulant (2.94% sodium citrate, 0.1 g/mL PGE1 and 1 U apyrase). Platelet-rich plasma was then obtained by centrifugation at 600 *g* for 15 min, and platelets were sedimented at 2000 *g* for 15 min. Platelet activation was induced with 1 U/ml collagen (Sigma-Aldrich) for 60 min at 37°C with gentle agitation. PMVs were then collected from the remaining supernatant with centrifugation at 20500 *g* for 90 min at 4°C as shown in the schematic diagrams (Supplementary Figure I; Bao et al., 2018). The obtained PMVs were quantified with NanoSight module (NanoSight NS300, United Kingdom) and 10^9^/mL PMVs were used for stimulation. The platelets treated with blank medium were used as the control group.

### Fluorescent Labeling of PMVs

PKH26 (MINI26, Sigma) was used to track PMVs according to the manufacturer’s protocol. Briefly, PKH26 prepared in Dilute C at a final concentration of 1 × 10^–6^ mol/L was used to incubate PMVs (final concentration was 1 × 10^7^ PMVs/mL) for 5 min at room temperature. The labeled PMVs (10^9^/mL) were incubated with VSMCs for 24 h. Then the samples were fixed with 4% paraformaldehyde for 15 min and counterstained with α-SMA-FITC antibody (Sigma, 1:500) for 2 h. Nuclei were stained with DAPI for 10 min. The images were obtained using a confocal laser scanning microscope (Fluoview 1000, Olympus).

### siRNA Transfection

For the RNA interference experiment, VSMCs were transfected with Lamtor1 siRNA or negative control siRNA (GenePharma, Shanghai, China) for 48 h with Lipofectamine 2000 in Opti-MEM (Thermo Fisher Scientific, Waltham, MA, United States) according to the manufacturer’s instructions. The sequences of siRNA oligos targeting Lamtor1 were: GCCG AGCC CAGC UACC AUAT T (5′–3′), UAUG GUAG CUGG GCUC GGCT T (5′–3′). The sequences of the negative control were: UUCU CCGA ACGU GUCA CGUT T (5′–3′), ACGU GACA CGUU CGGA GAAT T (5′–3′).

### Western Blot Analysis

Samples were lysed at 4°C for 10 min with RIPA lysis buffer containing 1 mM PMSF, and were centrifuged at 12000 *g* for 5 min. Then, the protein concentration in the supernatant was quantified with BCA kit (23227, Thermo Fisher). After being isolated by PAGE, the protein was transferred onto the polyvinylidene fluoride (PVDF) membrane and blocked with 5% no-fat milk at room temperature for 1 h. The membrane was subsequently incubated with diluted primary antibodies, respectively: phosphor-Src (Tyr416, 1:1000, CST), total Src (1:1000, CST), Lamtor1 (1:1000, CST), phosphor-p70S6K (Thr389, 1:1000, CST), phosphor-4E-BP1 (Thr37/46, 1:1000, CST), phosphor-mTORC1 (Ser2448, 1:1000, CST), total mTORC1 (1:1000, CST), α-SMA (1:1000, Proteintech), SM22 (1:1000, Proteintech), calponin (1:1000, Sigma), and GAPDH (1:5000, Proteintech). Protein bands were visualized by ECL kit (Beyotime) and the intensity was quantified by Quantity One (Bio-Rad).

### Ingenuity Pathway Analysis (IPA)

The Gene Ontology (GO) enrichment and Canonical Pathways of the published PMVs proteomic data (Dean et al., 2009) was analyzed by IPA software (Qiagen, content version: 39480507, https://www.qiagenbioinformatics.com/products/ingenuity-pathway-analysis). In addition, the possible molecules and transduction networks connected with Lamtor1 were obtained. IPA integrates the available knowledge on genes, drugs, chemicals, protein families, processes, and pathways, based on the interactions and functions derived from the Ingenuity Pathways Knowledge Database Literature (Dai et al., 2009).

### Statistics

All experiments were performed with at least three biological replicates, and the data were presented as the mean ± standard deviation (SD). Statistical analysis was performed using GraphPad Prism (version 8.1, GraphPad, San Diego, CA). Student’s *t* test was used for comparisons between two groups. *P* < 0.05 was regarded as statistically significant.

## Results

### PMVs Induce Lamtor1 Expression and Promote VSMC Dedifferentiation Both *in vivo* and *in vitro*

**FIGURE 1:**
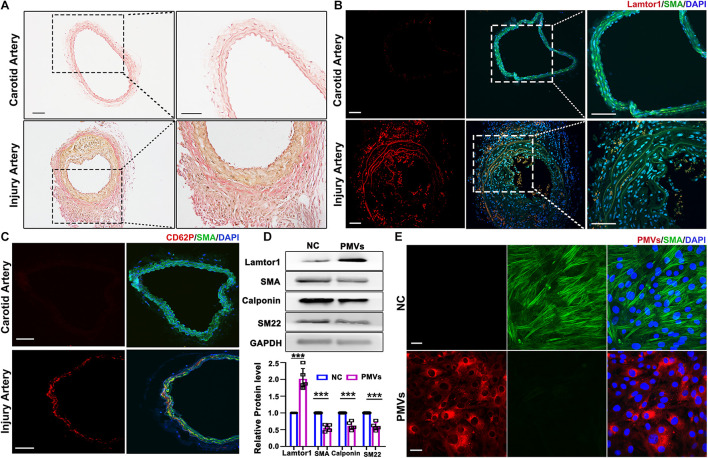
PMVs induced Lamtor1 expression and promoted VSMC dedifferentiation both *in vivo* and *in vitro*. **(A)** Representative images of Elastin Van-Gieson staining showed that the intimal hyperplasia was significantly thickened after 1-week wire injury in mouse model. Scale bars: 100 μm. **(B)** Representative images of immunostaining showed that Lamtor1 (red) was expressed in the injured intima after 1-week surgery. Green fluorescence was SMA, nuclei were counterstained with DAPI (blue). Scale bars: 100 μm. **(C)** Immunofluorescence staining showed the activated platelet signal labeled with CD62P (red) at the injury site 3 h after intimal injury surgery. Green fluorescence was SMA, nuclei were counterstained with DAPI (blue). Scale bars: 100 μm. **(D)** Western blot revealed that Lamtor1 expression was significantly increased and differentiated markers of VSMCs, including SMA, Calponin, and SM22 were decreased after incubating with PMVs for 24 h (*n* = 5). Data represent mean ± *SD*. *P* < 0.001. **(E)** Immunofluorescence staining indicated that PMVs (red) markedly decreased the expression of SMA (green) a differentiation marker. Nuclei were counterstained with DAPI (blue). Scale bars: 50 μm.

The carotid wire injury mouse model which mimics post-angioplasty restenosis in human was constructed to investigate the expression and function of Lamtor1 in the intimal hyperplasia. Elastin Van-Gieson staining showed that compared with the autologous artery control, the neointimal hyperplasia after 1-week injury was significantly thickened (Figure 1A), and the area of neointimal hyperplasia in the grafted vein was markedly increased (Supplementary Figure IIA). Immunofluorescence demonstrated that the expression of Lamtor1 was increased in the neointima (Figure 1B and Supplementary Figure IIB), and the co-expressed SMA, a contractile marker, was decreased (Figure 1B and Supplementary Figure IIC).

To explore the potential role of PMVs in VSMCs, the local adhesion of platelets at injured intima was then detected. Three hours after intimal injury, immunofluorescence staining showed that CD62P, the marker of activated platelet, was accumulated at the inner wall of injured blood vessels and contacted with the internal elastic membrane (Figure 1C and Supplementary Figure IID), which suggested the local activation of platelet after intimal injury. Furthermore, collagen I was used to activate platelets *in vitro* which simulated collagen exposure after vascular intimal injury *in vivo*, and the roles of PMVs in VSMC dedifferentiation and Lamtor1 expression were demonstrated.

Western blot revealed that protein expression of Lamtor1 was dramatically increased and the expressions of differentiation markers, i.e., SMA, calponin and SM22 were all significantly repressed in VSMCs treated with PMVs for 24 h compared with the negative control (Figure 1D).

To confirm the effect of PMVs on VSMC dedifferentiation, PMVs were labeled with PKH26, and treated VSMCs for 24 h. The immunofluorescence staining showed that PKH26 labeled PMVs adhered to VMSCs and markedly decreased the expression of SMA (Figure 1E).

These results suggested that neointima was obviously formed after carotid intimal injury, where Lamtor1 was increased and VSMCs were dedifferentiated, which may be caused by the PMVs released from the activated platelets.

### PMVs Promote VSMC Dedifferentiation via Lamtor1/mTORC1 Signaling

Lamtor1 has been reported to play a vital role in activating mTORC1 which controls cell growth and differentiation (Nowosad et al., 2020). Hence the responses of mTORC1 activation to Lamtor1 induced by PMVs were then investigated.

**FIGURE 2:**
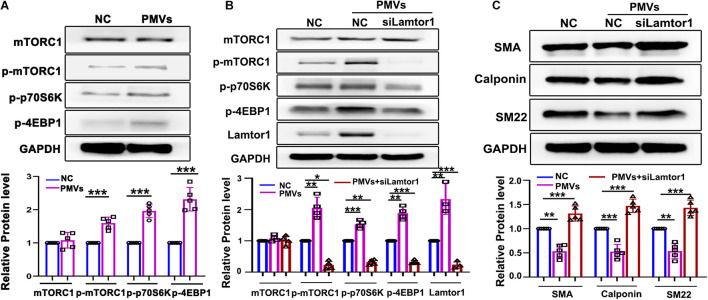
Specific knockdown of Lamtor1 reversed the effects of PMVs on mTORC1 activation and VSMC dedifferentiation. **(A)** Western blot showed that the phosphorylations of mTORC1, 4EBP1 and p70S6K in VSMCs were increased by PMVs, while the total mTORC1 level did not change (*n* = 5). Data represent mean ± SD. ***P < 0.001. **(B)** Specific siRNA transfection significantly reversed the effects of PMVs on expression of Lamitor1 and phosphorylations of mTORC1, 4EBP1 and p70S6K (*n* = 4). Data represent mean ± *SD.* *P < 0.05, **P < 0.01, ***P < 0.001. **(C)** Specific Lamtor1 siRNA increased the expressions of VSMC differentiation markers, including SMA, calponin and SM22 under PMV stimulation (*n* = 5). Data represent mean ± *SD.* **P < 0.01, ***P < 0.001.

Western blot results showed that the phosphorylations of mTORC1 and the main substrates of mTORC1, i.e., S6 kinase-1 (S6K1) which stimulates the initiation of translation (Magnuson et al., 2012), and eukaryotic translation initiation factor 4E (eIF4E)-binding protein-1 (4E-BP1) which promotes the release of 4E-BP from its inhibitory binding of eIF4E at the 5′-cap of mRNAs (Wang et al., 2019), were all significantly increased (Figure 2A).

To further investigate whether VSMC dedifferentiation induced by PMVs is dependent on Lamtor1, VSMCs were transfected with small interfering RNA (siRNA) specifically targeting at Lamtor1 and then treated with PMVs. Western blot results indicated that the Lamtor1 expression level was significantly suppressed by Lamtor1 siRNA in comparison with the negative control group (Figure 2B). The increased expressions of p-mTORC1 and the substrates, i.e., p-p70S6K and p-4EBP1 induced by PMVs was markedly reversed by Lamtor1 siRNA transfection (Figure 2B). Furthermore, the VSMC differentiation revealed similar negative correlation with Lamtor1 expression (Figure 2C).

### PMVs Induce Phosphorylation of Src

**FIGURE 3:**
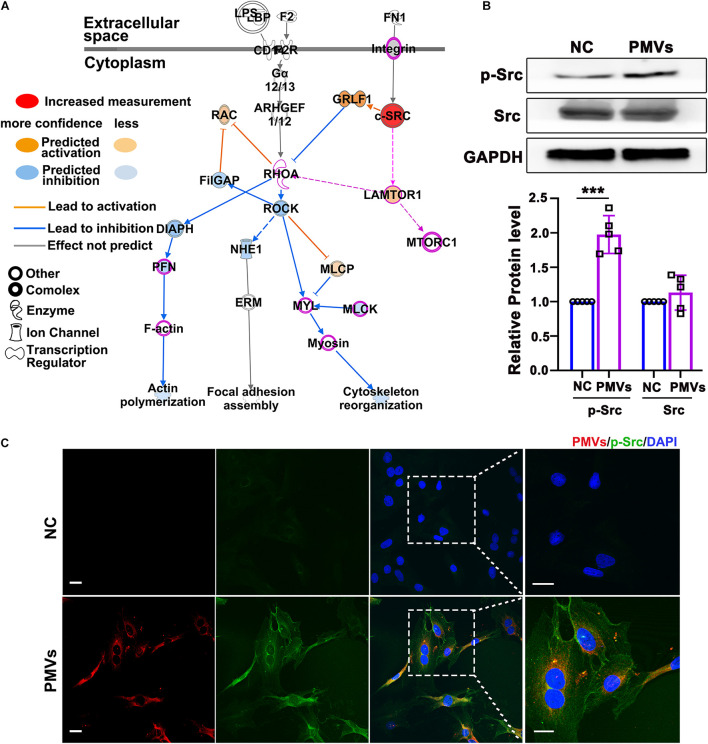
PMVs induced Src phosphorylation. **(A)** Based on previously published proteomic data of PMVs, IPA bioinformatic analysis indicated that PMVs may induce Src activation, which may be the potential upstream molecule of Lamtor1 and participated in VSMC dedifferentiation. Solid lines for direct association and dotted lines for indirect or predicted association. **(B)** Western blot revealed that PMVs significantly increased the phosphorylation of Src, while did not change the expression of total Src (*n* = 5). Data represent mean ± *SD*. ***P < 0.001. **(C)** Immunofluorescence staining indicated that the expression of p-Src (green) was significantly increased after incubated with PMVs (red). Nuclei were counterstained with DAPI (blue). Scale bars: 50 μm.

To explore the possible mechanism by which PMVs regulated Lamtor1 expression and VSMC dedifferentiation, Ingenuity Pathway Analysis (IPA, QIAGEN, Dusseldorf, Germany) was used to analyze the proteomic data of PMVs released by activated platelets which has been published in NCBI database (Dean et al., 2009; Figure 3A). Gene Ontology analysis revealed that 556 proteins expressed in PMVs were enriched into a series of important biological processes (Supplementary Figure III). Ingenuity Canonical Pathways Analysis revealed that these 556 proteins mainly contributed to actin cytoskeleton signaling and integrin signaling (Supplementary Figure IV). Further pathway analysis revealed that Src may be the upstream molecule of Lamtor1 and participate in actin cytoskeleton in differentiation (Figure 3A).

Western blot was used to confirm the regulating effect of PMVs on Src expression or activation in VSMCs, and the result showed that the phosphorylation of Src was significantly increased by PMV treatment (Figure 3B), meanwhile the total level of Src was not changed (Figure 3B). The immunofluorescence staining verified the increased phosphorylation of Src in cytoplasm of VSMCs (Figure 3C).

### Src Phosphorylation Participates in VSMC Dedifferentiation, Lamtor1 Expression and mTORC1 Activation Induced by PMVs

**FIGURE 4:**
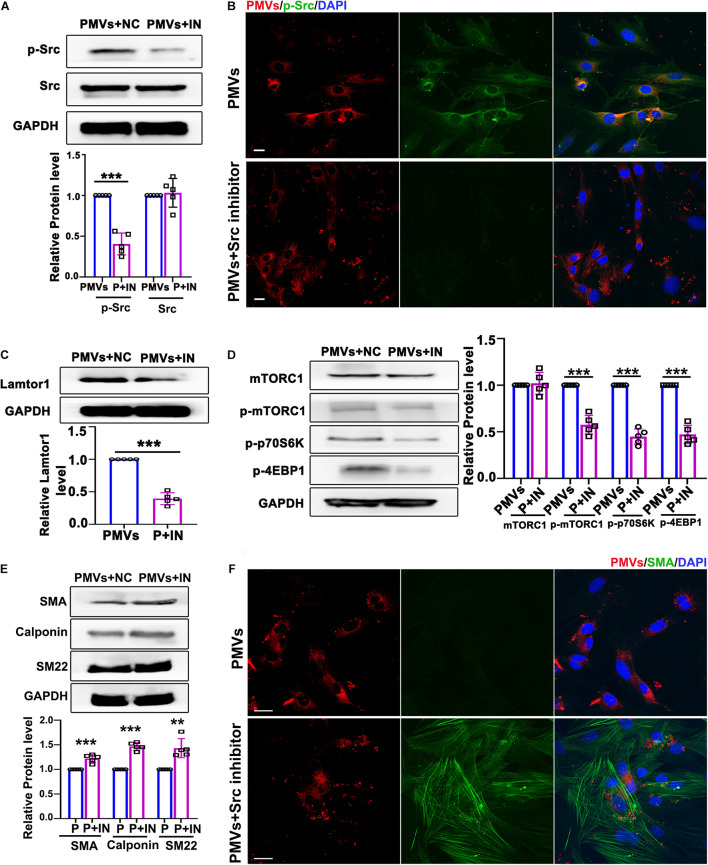
Inhibition of p-Src repressed VSMC dedifferentiation *via* decreasing Lamtor1 expression and mTORC1 activity. **(A)** Src inhibitor (IN) decreased the phosphorylation of Src induced by PMVs, while did not change the expression of total Src (*n* = 5). Data represent mean ± *SD*. ***P < 0.001. **(B)** Immunofluorescence staining confirmed that the phosphorylation of Src (green) was decreased after incubated with Src IN. Nuclei were counterstained with DAPI (blue). Scale bars: 50 μm. **(C)** Src IN suppressed the protein expression of Lamtor1 induced by PMV stimulation (*n* = 5). Data represent mean ± *SD*. ***P < 0.001. **(D)** Src IN inhibited phosphorylations of mTORC1, 4EBP1 and p70S6K induced by PMVs, while the expression of total mTORC1 was not changed (*n* = 5). Data represent mean ± *SD*. ***P < 0.001. **(E)** Western blot revealed that Src IN increased the expressions of VSMC differentiation markers, i.e., SMA, calponin and SM22, (*n* = 5). Data represent mean ± *SD.* **P < 0.01, ***P < 0.001. **(F)** Immunofluorescence staining confirmed that Src inhibitor increased the expression of SMA (green). Nuclei were counterstained with DAPI (blue). Scale bars: 50 μm.

Data described above suggested that PMVs phosphorylated Src and induced VSMC dedifferentiation. To confirm whether the effect of PMVs on VSMC dedifferentiation and Lamtor1 expression was Src dependent, specific Src inhibitor was used to pre-incubate VSMCs 1 h before PMV stimulation. Western blot result showed that Src inhibitor significantly reversed the phosphorylation of Src induced by PMVs (Figure 4A), but there was no significant change in the expression of total Src (Figure 4A). Immunofluorescence staining confirmed the decreased phosphorylation of Src in cytoplasm of VSMCs (Figure 4B).

Western blot also showed that Lamtor1 expression was significantly decreased by pretreatment of specific Src inhibitor (Figure 4C). The phosphorylations of mTORC1 and its substrates 4E-BP1 and p70S6K were all repressed by Src inhibitor (Figure 4D) under PMVs treatment. Moreover, Src inhibitor promoted the expression of VSMC differentiation markers, i.e., SMA, calponin, and SM22 (Figure 4E), and immunofluorescence staining also showed that the contractile fibers of SMA were increased dramatically (Figure 4F). Whereas, Src inhibitor revealed no significant effect on the adherent level of PMVs to VSMCs (Supplementary Figure V).

These data demonstrated that Src phosphorylation, in response to PMV treatment, modulated Lamtor1 expression and mTORC1 activation, which subsequently promoted VSMC dedifferentiation *in vitro*.

### SMC-Specific *Lamtor1* KO Inhibits mTORC1 Activation and Represses Intima Hyperplasia *in vivo*

**FIGURE 5:**
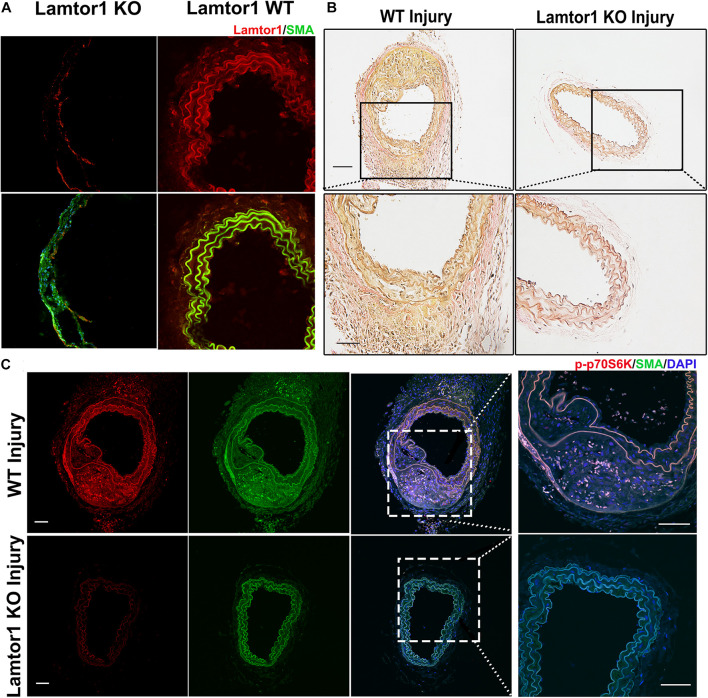
SMC-specific *Lamtor1* KO repressed mTORC1 activation and intima hyperplasia after injury. **(A)** Representative images of immunostaining showed that in SMC-specific *Lamtor1* KO mice, Lamtor1 (red) was rarely expressed in VSMCs which was stained with SMA (green) in the wire injured carotid artery after 1-week surgery. Scale bars: 20 μm. **(B)** Representative images of Elastin Van-Gieson staining revealed that neointimal formation was significantly reduced in SM-specific *Lamtor1* KO mice after 1-week surgery. Scale bars: 100 μm. **(C)** Representative images of immunostaining showed that p-p70S6K (red) was greatly repressed in the wire injured carotid artery of SMC-specific *Lamtor1* KO mice after 1-week surgery. Nuclei are counterstained with DAPI (blue). Scale bars: 20 μm.

To assess the role of Lamtor1 in neointimal formation after intimal injury, SMC-specific *Lamtor1* KO mice combined with intimal injury was generated. Immunostaining revealed that the expression of Lamtor1 was abolished in the media layer of carotid artery of SMC-specific *Lamtor1* KO mice compared with littermate control (Figure 5A). SMC-specific *Lamtor1* KO significantly reduced the neointima after intimal injury for 1 week compared to the littermate wild type (WT) mice (Figure 5B). Meanwhile, immunostaining revealed a decrease of p-p70S6K in SMC-specific *Lamtor1* KO mice after intimal injury in comparison with WT mice (Figure 5C). These data suggested that specific repression of Lamtor1 in VSMCs *in vivo* attenuated mTORC1 signaling activation and may contribute to neointimal formation after intimal injury.

## Discussion

Our present study demonstrated that Lamtor1 in VSMC was crucial in intimal remodeling and its repressed expression markedly attenuated intima hyperplasia after carotid injury. It has been reported that Lamtor1 suppresses the activation of mTORC1 on the lysosomal surface, and subsequently regulates cell growth and homeostasis *via* various signaling pathways (Bar-Peled et al., 2012). For example, Lamtor1 interacts with p27 in lysosomes during starvation and prevents mTORC1 activation, which promotes autophagy (Nowosad et al., 2020). Nada S et al. revealed that Lamtor1-mTORC1 is involved in the macromolecule biosynthesis, which contributes to autophagy and cell growth (Nada et al., 2009). Furthermore, dysregulation of mTORC1 signaling pathway occurs in Alzheimer’s disease and cancer (Laplante and Sabatini, 2012). In cardiovascular system, loss of mTOR activity represses endothelial proliferation and angiogenesis as well as the proliferation of endothelial progenitor cells, which limits tissue repair and regeneration after cardiac injury (Humar et al., 2002; Miriuka et al., 2006). Rapamycin, the specific inhibitor of mTORC1, effectively reduces restenosis by inhibiting the growth of VSMCs and has been approved by the FDA for clinical application after angioplasty (Stefanini and Holmes, 2013). Additionally, our present study suggested that Lamotr1 may also be a potential target for neointima formation after injury. It has been reported that complex cell responses are induced after vessel injury. The rapid proliferation and migration of endothelial cells are necessary for the repair of blood vessel damage, while the consistent proliferation, migration and dedifferentiation of VSMCs contribute to the intimal hyperplasia and the long-term patency (Tesfamariam, 2016). How to spatial and temporal adjust Lamtor1 to help blood vessel reconstruction is very important and interesting, which needs more researches in the future.

Recently, Block et al. (2020) revealed that Lamtor1 regulates Src localization on late endosome, and correlates Src trafficking toward the plasma membrane specifically at focal adhesion sites, which facilitates YAP nuclear translocation and activation. Furthermore, silencing Lamtor1 dephosphorylates Src at Tyr-416 thus attenuates Src catalytic activity (Block et al., 2020). Interestingly, in our present study, based on previously published proteomic data of PMVs, IPA bioinformatics analysis indicated that Src may also act as upstream molecule of Lamtor1.

The protein tyrosine kinase Src is a highly homologous prototype of non-receptor type tyrosine kinases, which ubiquitously expresses in various cell types and is intimately involved in many diseases, such as oncogenesis, cardiovascular diseases, and so on (Li et al., 2020). Phosphorylation of Src at tyrosine 419 is crucial to full kinase activation (Chiang and Sefton, 2000), which plays important roles in cellular proliferation, survival, adhesion and migration.

In cardiovascular system, Src kinase is highly expressed in VSMCs, endothelial cells, and myocytes (Kim et al., 2009; Wang and Aikawa, 2015), and its activity is associated with the physiological and pathological status of cells. In vascular morphogenesis, Src kinase plays critical roles in creating and decorating the endothelial cell apical membrane surface during early and late stages of lumen and tube formation (Kim et al., 2017). Inhibition of Src activity reduces oxidative stress, improves endothelial function, and normalizes ERK1/2 signaling hyper-activation, which result in attenuation of hypertension development (Callera et al., 2016). It has been reported that Src mediates Ang-II dependent VSMC proliferation through the ERK2 and/or Ca^2+^ signaling in pathological conditions (Touyz et al., 2001; Sayeski and Ali, 2003). All these studies have demonstrated a key role of Src in promoting proliferation and migration of VSMC, while our results further indicate that Src was activated by PMVs secreted from recruited and activated platelets at injured intima, which subsequently promoted VSMC dedifferentiation *via* Lamtor1/mTORC1 pathway.

In normal adult, VSMCs maintains a contractile phenotype which proliferates slowly and expresses a range of contractile proteins for functional contraction. In response to vascular injury or local environmental alteration, contractile VSMCs dedifferentiate to synthetic phenotype (Allahverdian et al., 2018), characterized as repressed contractile proteins, altered morphology from elongated/spindle-like to thomboid/epitheloid-like, increased proliferation, and facilitated migration (Li et al., 2017). In healthy vascular wall, individual VSMC dedifferentiation occurs at a low event rate which participates in vascular wall repair. However, persistent pathological VSMC dedifferentiation significantly contributes to cardiovascular disease, such as atherosclerosis, hypertension, or graft failure (Owens et al., 2004). Therefore, VSMC phenotypic switch is an important step which leads to vascular remodeling. However, the mechanisms underlying VSMC dedifferentiation in intimal injury are poorly understood. It has been reported that inhibition of mTORC1stabilized GATA-6, which then activates transcription of promoters encoding contractile proteins, represses VSMC dedifferentiation (Xie et al., 2015). Our study suggested an important role of PMVs in VSMC dedifferentiation *via* modulating Lamtor1 expression and mTORC1 activation. Interestingly, immunofluorescent result of Figure 1E showed that although some VSMCs adhered less PMVs, the cell dedifferentiation was still strong, which suggested that after reach an appropriate range, PMVs probably revealed a similar regulation on VSMC functions. Since VSMC dedifferentiation is a long-term effect after PMV adhesion and PMVs adhesion may trigger a series of continuous and amplified signaling cascade, the efficient concentration of PMVs on VSMC functions needs systematic researches.

IPA analysis also predicts that PMVs may trigger the integrins family on the membrane of VSMCs which then modulate Src activation (Figure 3A and Supplementary Figure IV). It will be intriguing to examine the mechanisms by which PMVs modulate integrins on the membrane of VSMCs in the recent future.

**FIGURE 6:**
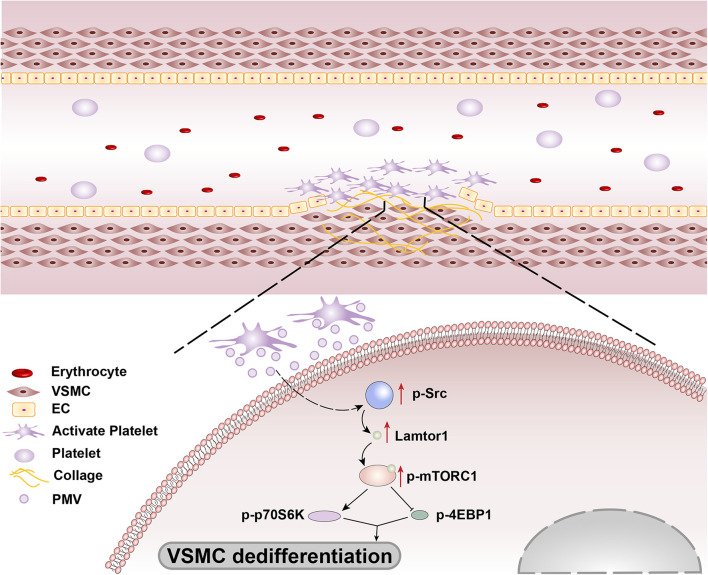
Schematic drawing of the mechanisms of Lamtor1 in intimal hyperplasia after intimal injury.

In summary, our findings revealed that the platelets are recruited and activated at the injured intima and then secrete bulk of PMVs. The PMVs significantly phosphorylated Src in VSMCs to promote Lamtor1 expression and activate mTORC1 signaling pathway, thus induced VSMC dedifferentiation (Figure 6). Intriguingly, SMC-specific *Lamtor1* KO obviously attenuated intima hyperplasia in injury model, which suggested that Lamtor1 and the related molecules may provide potential therapeutic targets to ameliorate intimal hyperplasia after vascular interventional surgery.

## Data Availability Statement

The original contributions presented in the study are included in the article/Supplementary Material, further inquiries can be directed to the corresponding author/s.

## Ethics Statement

The animal study was reviewed and approved by Animal Research Committee of Shanghai Jiao Tong University.

## Author Contributions

Y-XQ and J-TL designed and guided the work. J-TL and HB participated in data collection, data processing, program implementation, and manuscript writing. Y-JF and Z-TL contributed to statistical analysis. Y-XQ, Q-PY, and M-LZ contributed to manuscript writing and article publication. Z-LJ and YH revised the manuscript critically. Y-XQ and Z-LJ provided funding acquisition. All authors provided critical advice for the final manuscript.

## Conflict of Interest

The authors declare that the research was conducted in the absence of any commercial or financial relationships that could be construed as a potential conflict of interest.

## Publisher’s Note

All claims expressed in this article are solely those of the authors and do not necessarily represent those of their affiliated organizations, or those of the publisher, the editors and the reviewers. Any product that may be evaluated in this article, or claim that may be made by its manufacturer, is not guaranteed or endorsed by the publisher.
