# Supplementary material for: Platelet-Derived Microvesicles Promote VSMC Dedifferentiation After Intimal Injury via Src/Lamtor1/mTORC1 Signaling
Source: Front Cell Dev Biol. 2021 Sep 16;9:744320. doi: 10.3389/fcell.2021.744320 (PMC8481604; doi:10.3389/fcell.2021.744320)
Supplement: Supplementary file 1 [file Table_1.docx]

Supplemental Materials for

**Platelet-derived microvesicles promote VSMC dedifferentiation after intimal injury via Src/Lamtor1/mTORC1 signaling**

**Supplemental Figures**


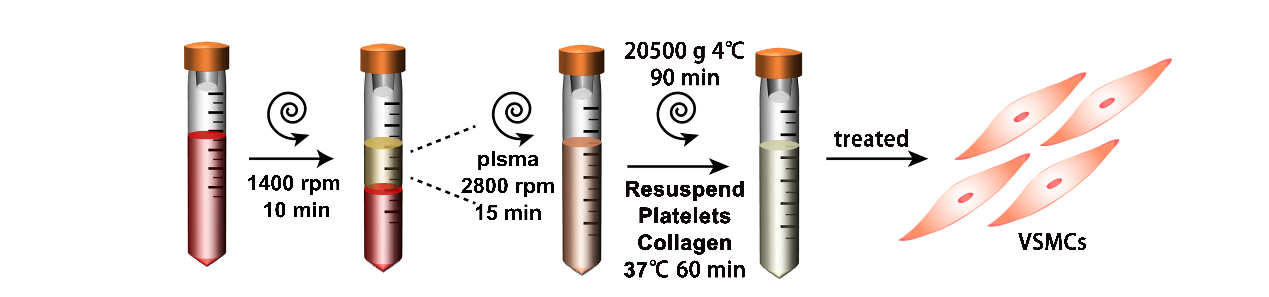


**Supplemental Figure I. Schematic diagrams showed the method used to obtain PMVs from collagen activated platelets.**


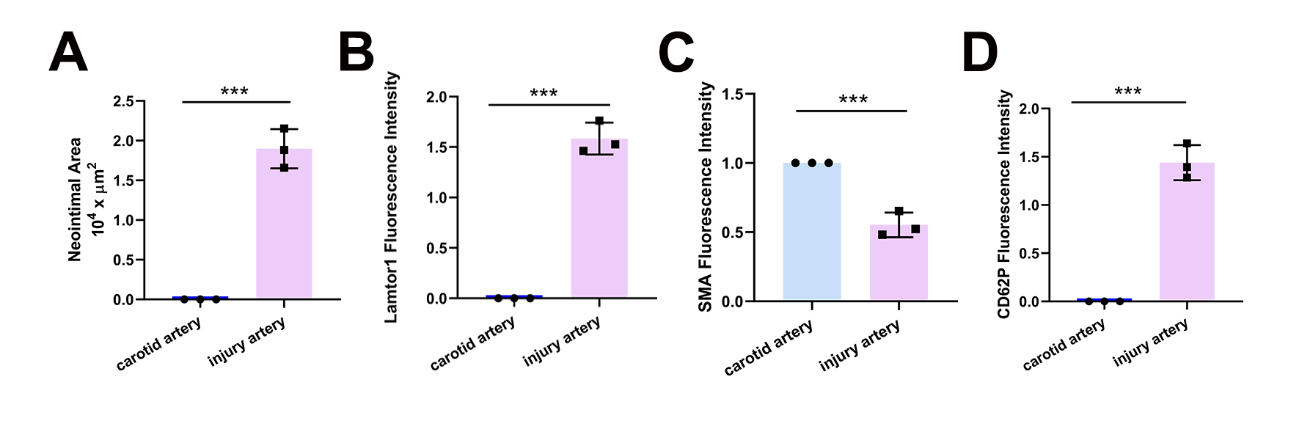


**Supplemental Figure II. Quantitative analysis of Fig1 in the manuscript.** A. Quantification of the intimal area in Fig1A. B. Quantification of Lamtor1 fluorescence intensity in Fig1B. C. Quantification of SMA fluorescence intensity in Fig1B. D. Quantification of CD62P fluorescence intensity in Fig1C. Each dot represents a single mouse. Values are represented as mean ± SD.


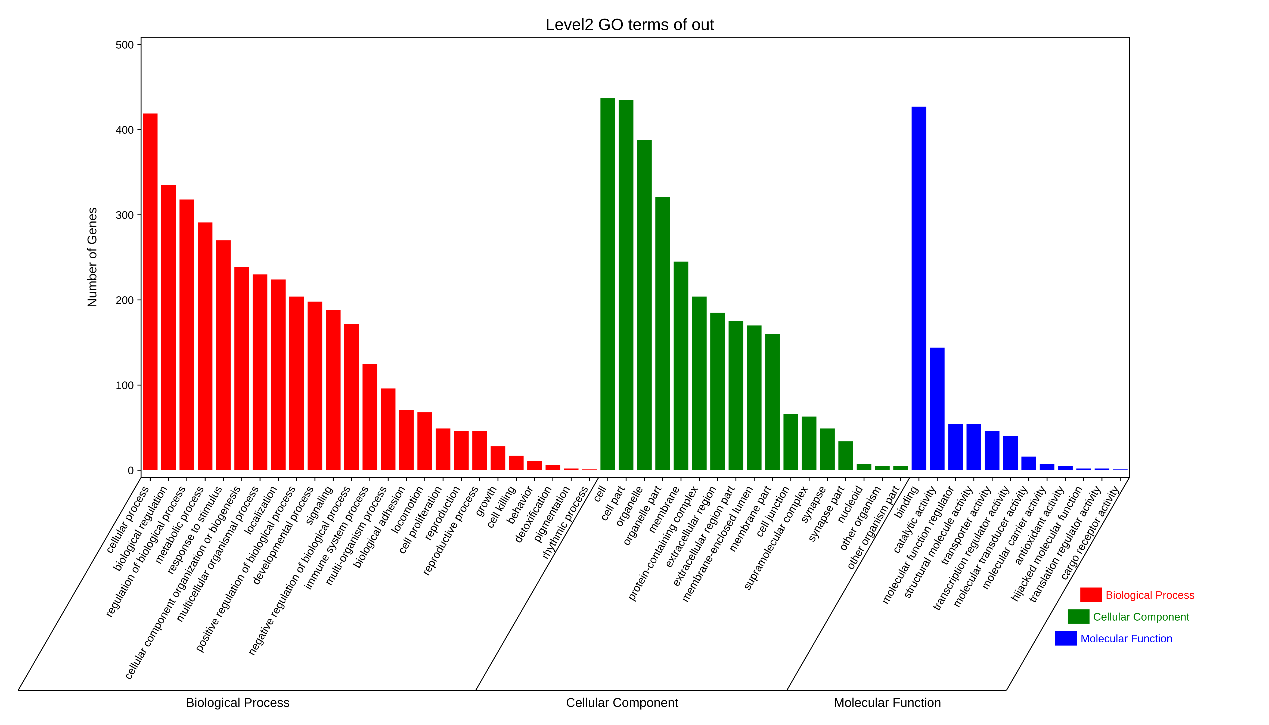


**Supplemental Figure III. The Gene Ontology (GO) annotation of proteins expressed in PMVs analyzed by IPA.** 556 proteins expressed in PMVs were classified into three main GO categories (cellular components, molecular functions and biological processes). The y-axis showed the number of genes in the same category.


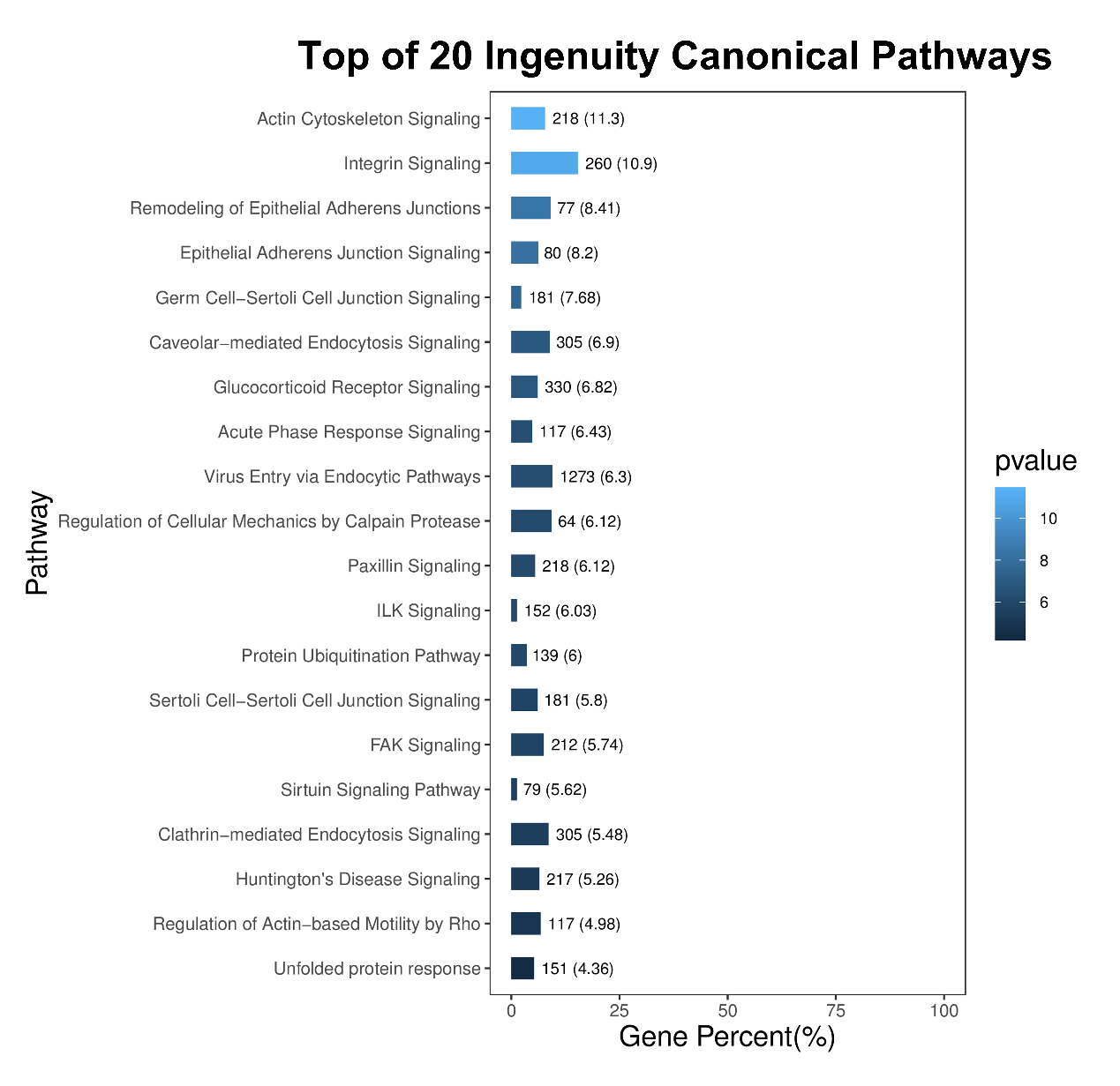


**Supplemental Figure IV. The top 20 ingenuity canonical pathways analyzed by IPA based on PMV proteomic data.** 556 proteins expressed in PMVs were analyzed by IPA, and top 20 pathways were presented.


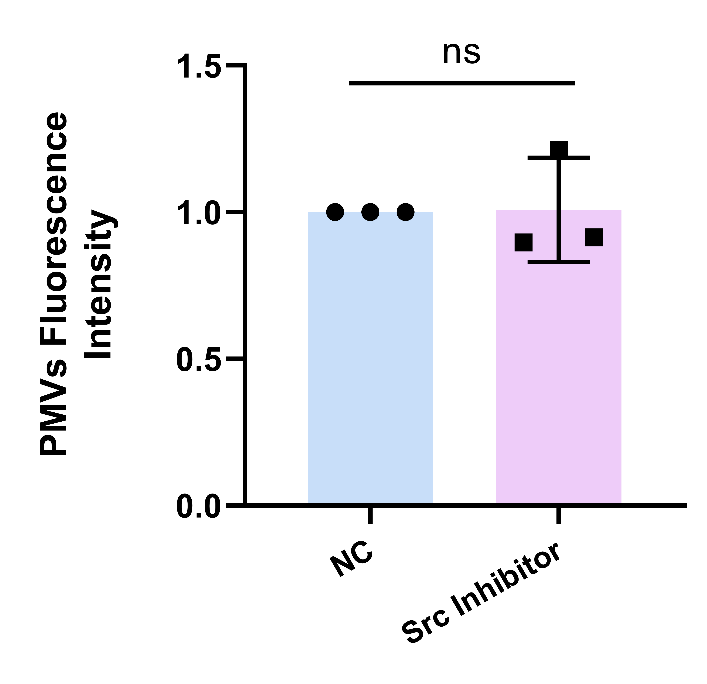


**Supplemental Figure V. Quantitative analysis of the fluorescence intensity of PMVs in Fig4F in the manuscript.** Compared with negative control (NC), Src inhibitor had no significant effect on the adherent level of PMVs to VSMCs. Each dot represents a single mouse. Values are represented as mean ± SD.
