# Supplementary material for: Platelet-Derived Microvesicles Promote VSMC Dedifferentiation After Intimal Injury via Src/Lamtor1/mTORC1 Signaling
Source: Front Cell Dev Biol. 2021 Sep 16;9:744320. doi: 10.3389/fcell.2021.744320 (PMC8481604; doi:10.3389/fcell.2021.744320)

Original data for "Platelet-derived microvesicles promote VSMC dedifferentiation after intimal injury via Src/Lamtor1/mTORC1 signaling"

1. Full unedited gel for Figure 1D-Lamtor1.

(1) Full unedited gel for Figure 1D-Lamtor1 presented in the manuscript.

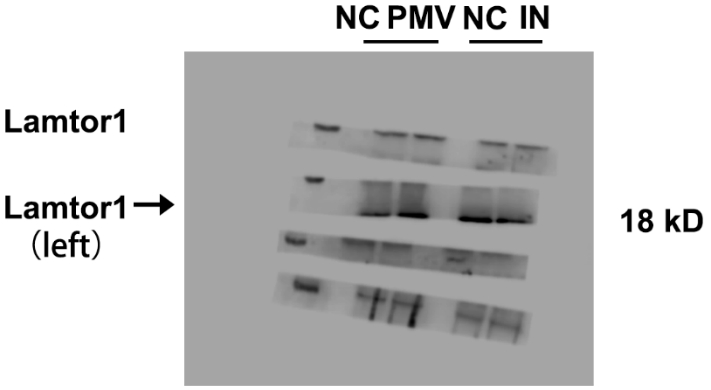

(2) Full unedited gel of Fig1D-Lamtor1 for all replication.

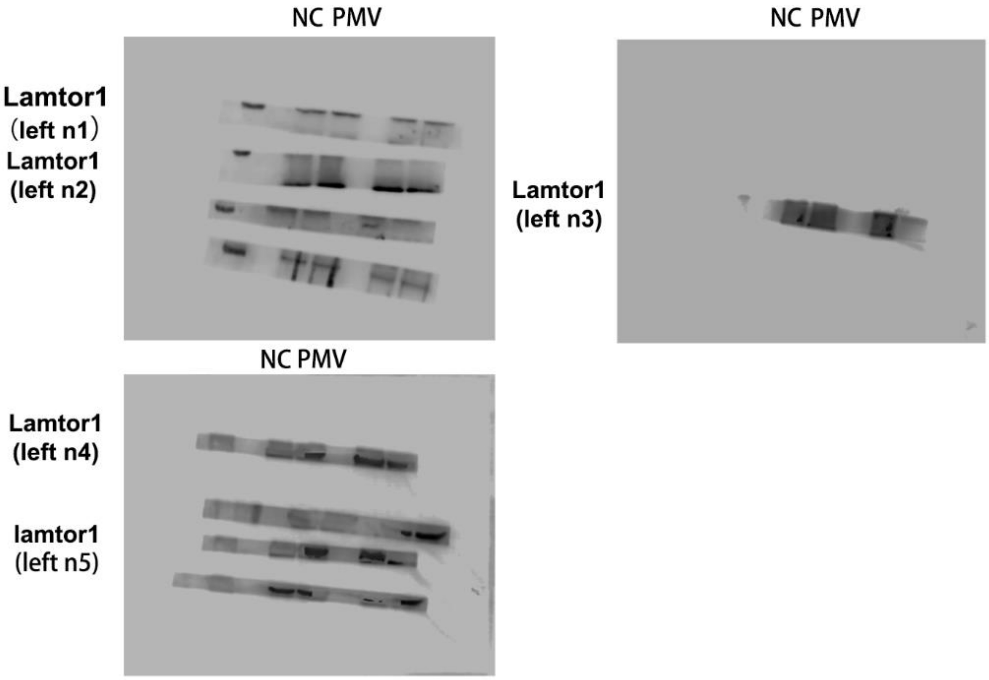

2. **Full unedited gel for Figure 1D-SMA.**

(1) Full unedited gel for Figure 1D-SMA presented in the manuscript.

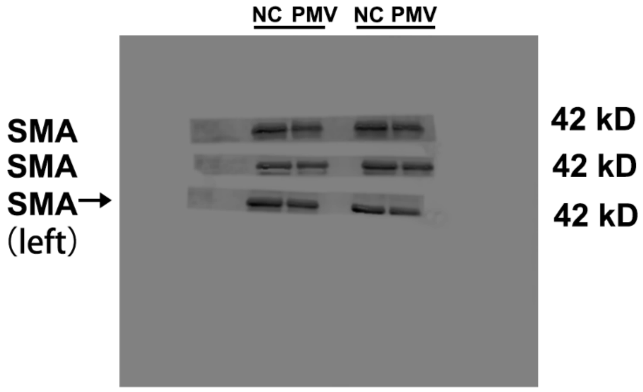

(2) Full unedited gel of Fig1D-SMA for all replication.

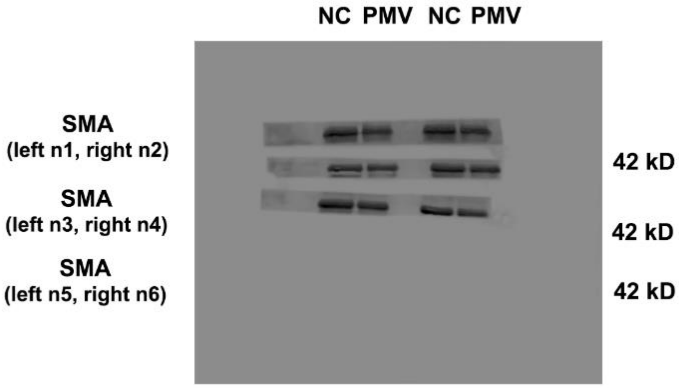

3. **Full unedited gel for Figure 1D-Calponin.**

(1) Full unedited gel for Figure 1D-Calponin presented in the manuscript.

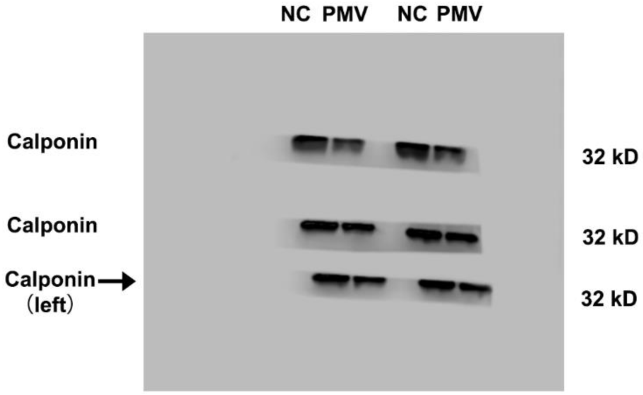

(2) Full unedited gel of Fig1D-Calponin for all replication.

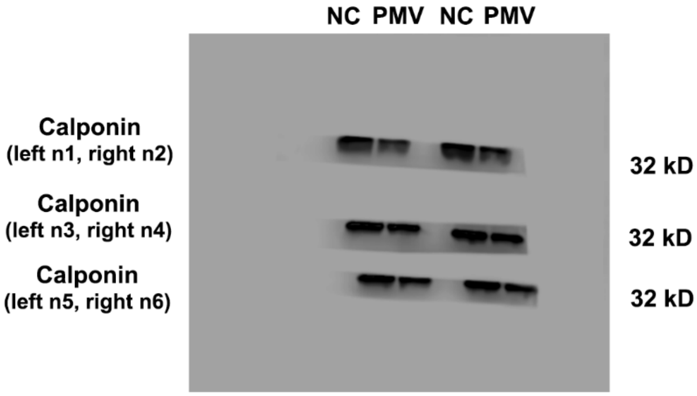

4. **Full unedited gel for Figure 1D-SM22 and GAPDH.**

(1) Full unedited gel for Figure 1D-SM22 and GAPDH presented in the manuscript.

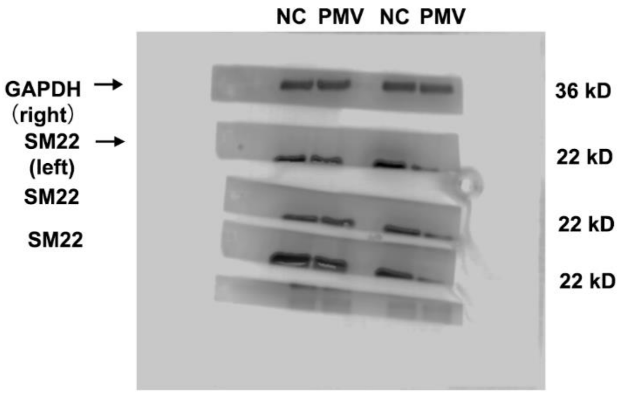

(2) Full unedited gel of Fig1D- SM22 and GAPDH for all replication.

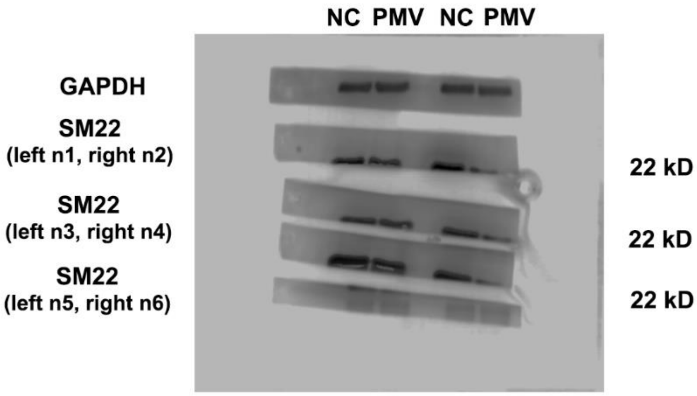

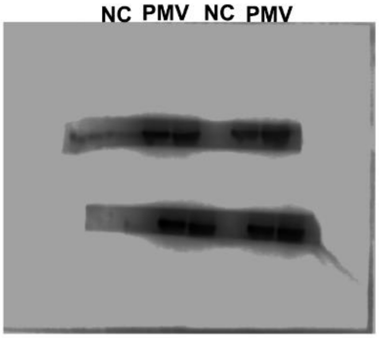

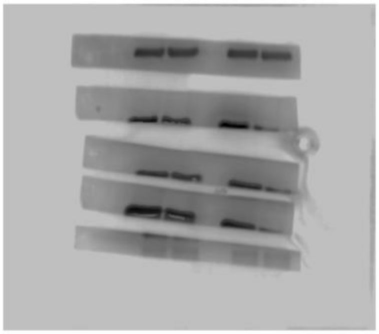

5. Full unedited gel for Figure 2A-mTORC1.

(1) Full unedited gel for Figure 2A-mTORC1 presented in the manuscript.

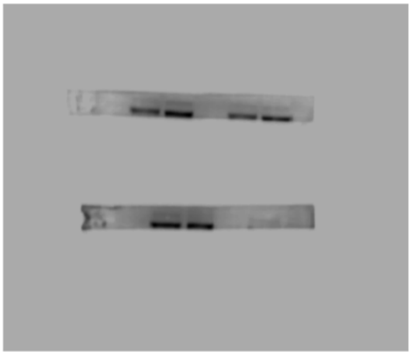

(2) Full unedited gel of 2A-mTORC1 for all replication.

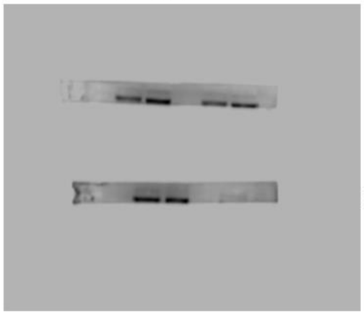

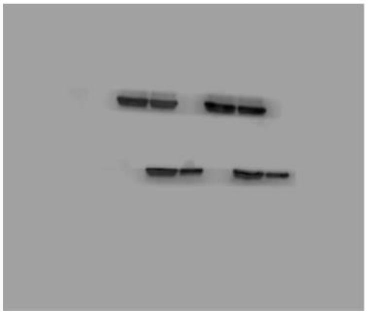

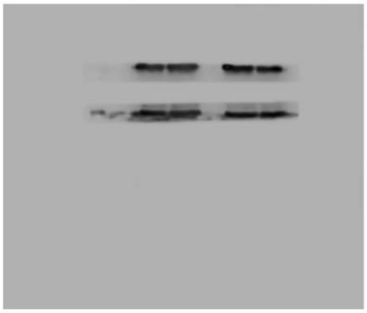

6. Full unedited gel for Figure 2A-p-mTORC1.

(1) Full unedited gel for Figure 2A-p-mTORC1 presented in the manuscript.

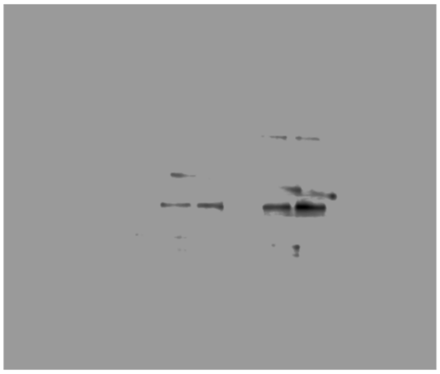

(2) Full unedited gel of Fig2A-p-mTORC1 for all replication.

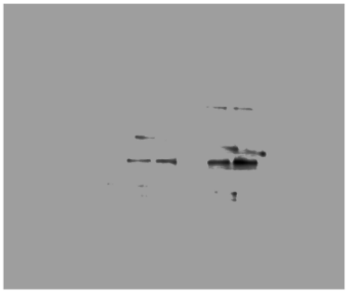

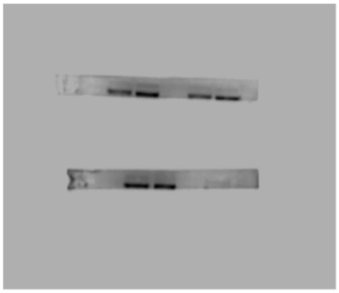

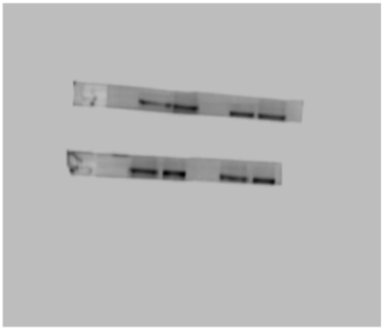

7. Full unedited gel for Figure 2A-p-p70S6K.

(1) Full unedited gel for Figure 2A-p-p70S6K presented in the manuscript.

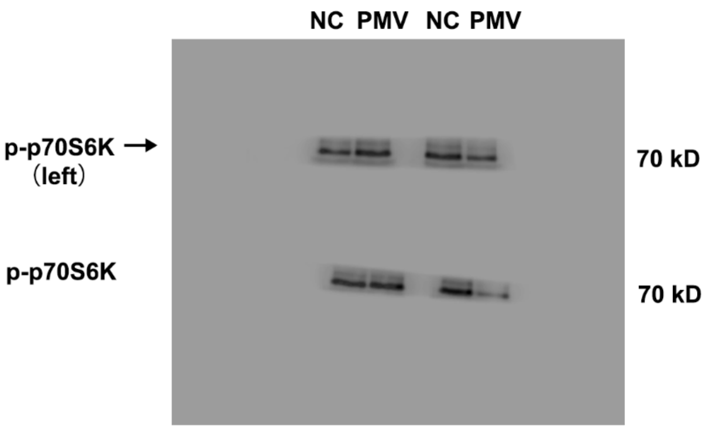

(2) Full unedited gel of Fig2A-p- p70S6K for all replication.

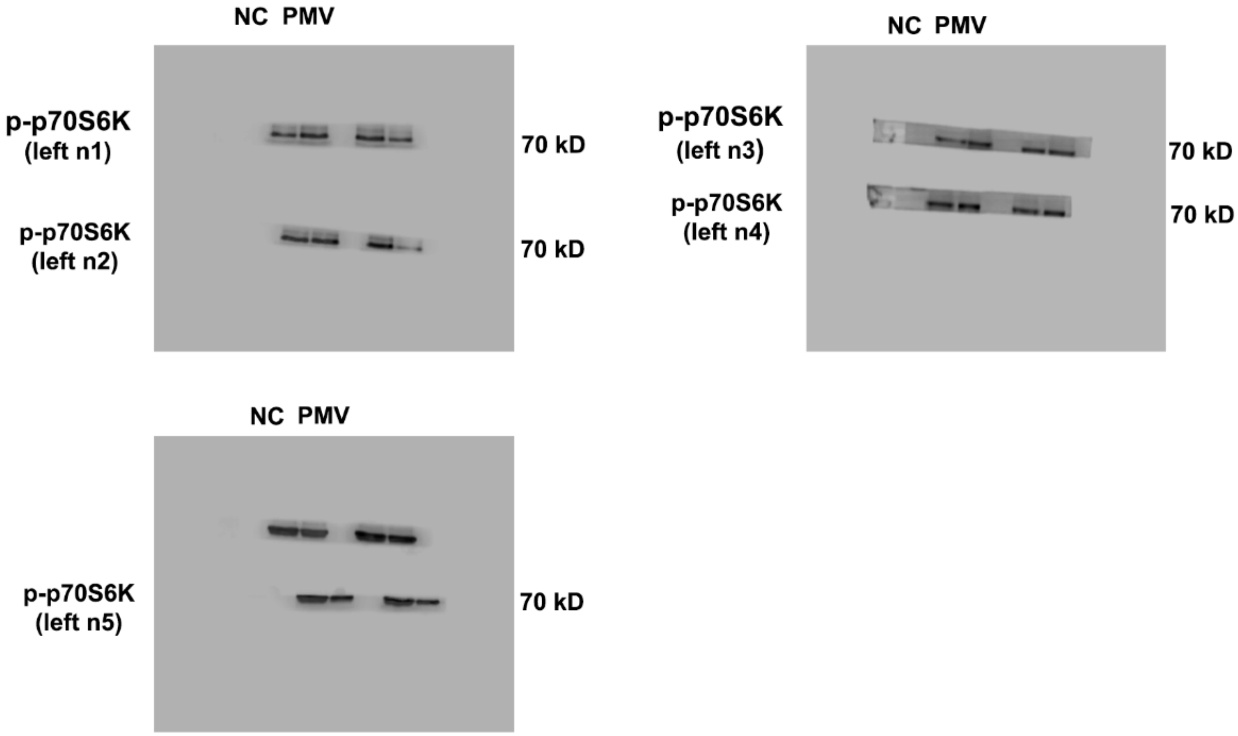

8. Full unedited gel for Figure 2A-p-4EBP1.

(1) Full unedited gel for Figure 2A-p-4EBP1 presented in the manuscript.

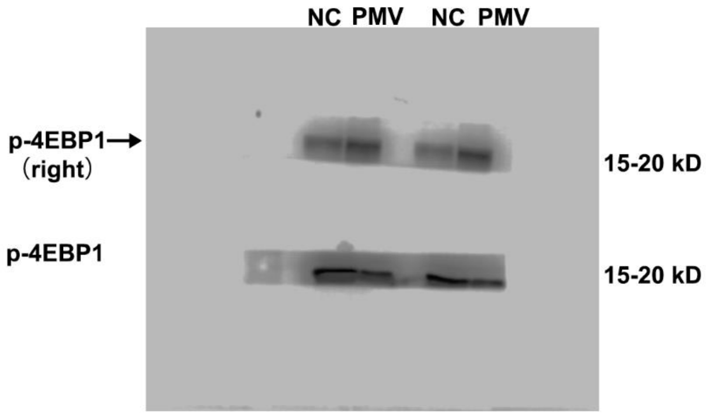

(2) Full unedited gel of Fig2A- p-4EBP1 for all replication.

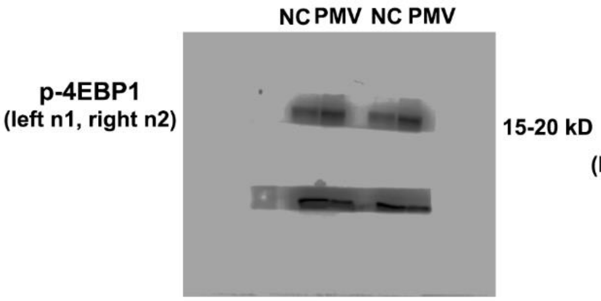

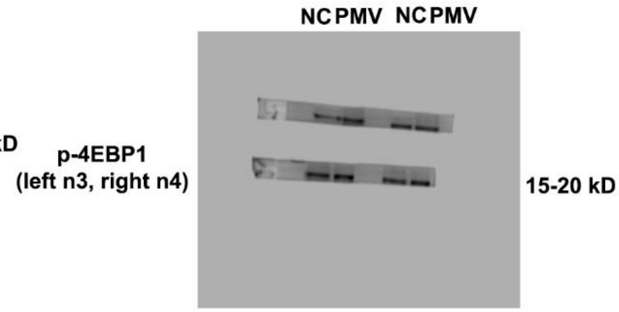

9. Full unedited gel for Figure 2A-GAPDH.

(1) Full unedited gel for Figure 2A-GAPDH presented in the manuscript.

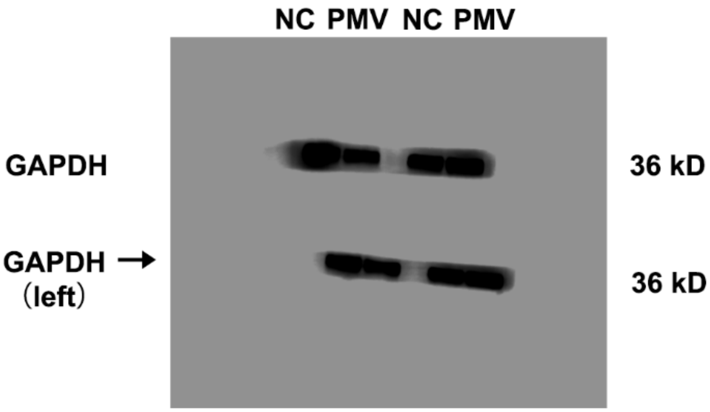

(2) Full unedited gel of Fig2A- GAPDH for all replication.

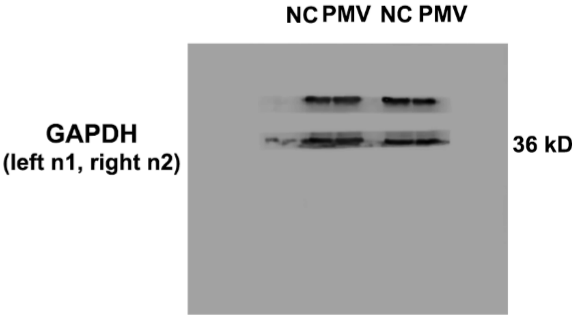

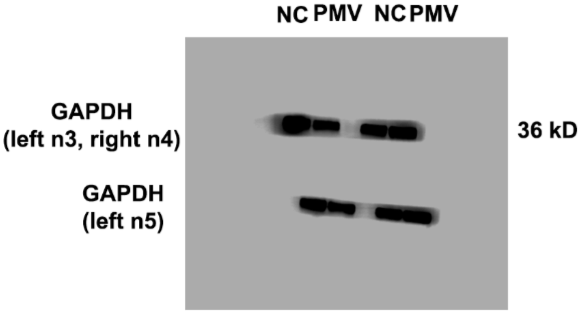

10. Full unedited gel for Figure 2B-mTORC1.

(1) Full unedited gel for Figure 2B-mTORC1 presented in the manuscript.

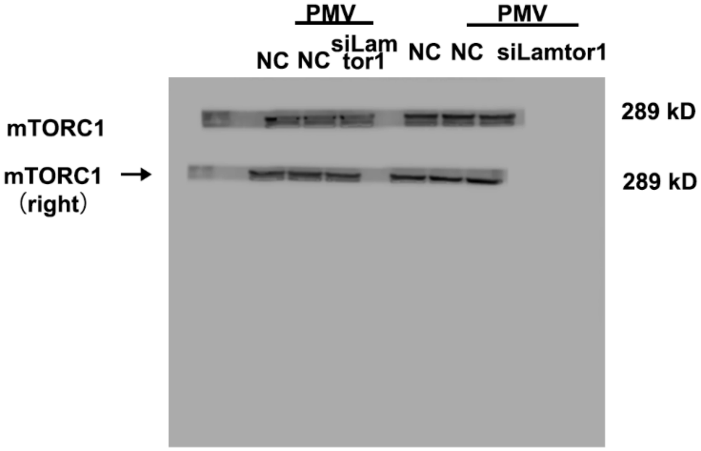

(2) Full unedited gel of Figure 2B-mTORC1 for all replication.

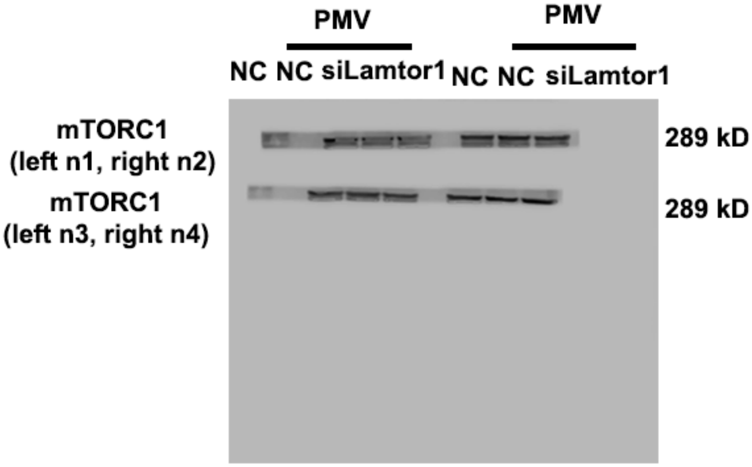

11. Full unedited gel for Figure 2B-p-mTORC1 and Lamtor1.

(1) Full unedited gel for Figure 2B-p-mTORC1 and Lamtor1 presented in the manuscript.

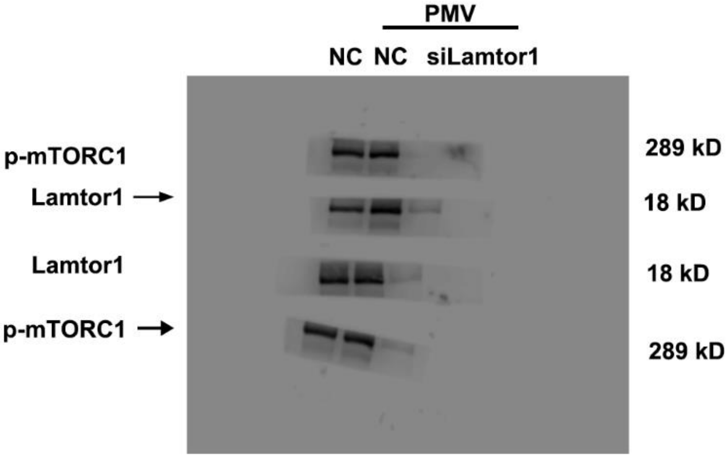

(2) Full unedited gel of Figure 2B- p-mTORC1 for all replication.

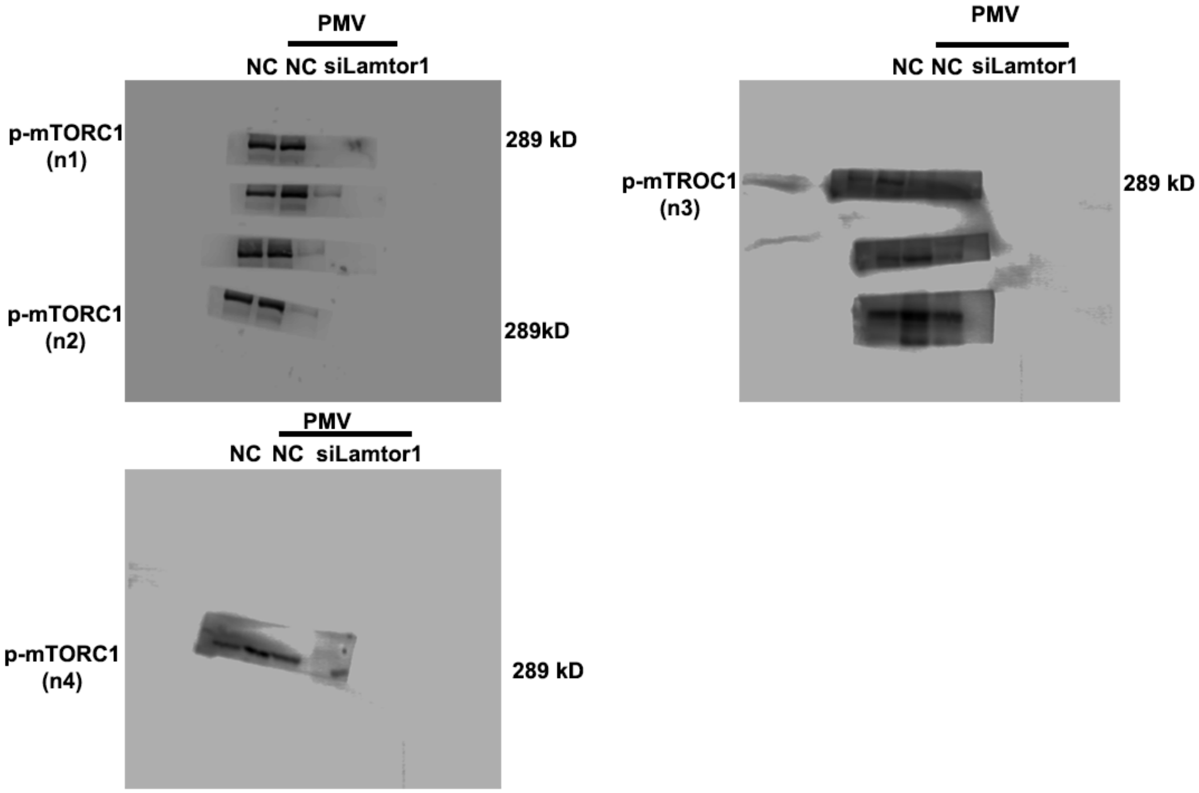

12. Full unedited gel for Figure 2B-p-p70S6K and p-4EBP1 Lamtor1.

(1) Full unedited gel for Figure 2B-p-p70S6K and p-4EBP1 Lamtor1 presented in the manuscript.

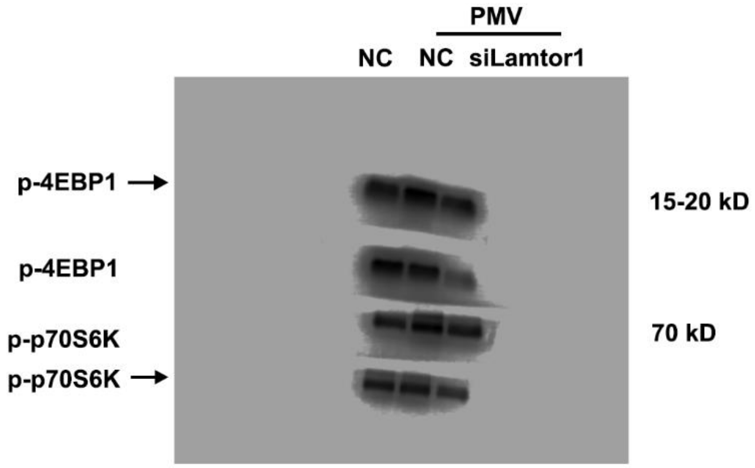

(2) Full unedited gel of Figure 2B- p-p70S6K and p-4EBP1 for all replication.

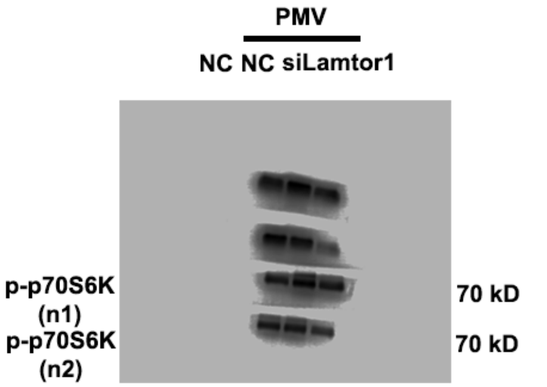

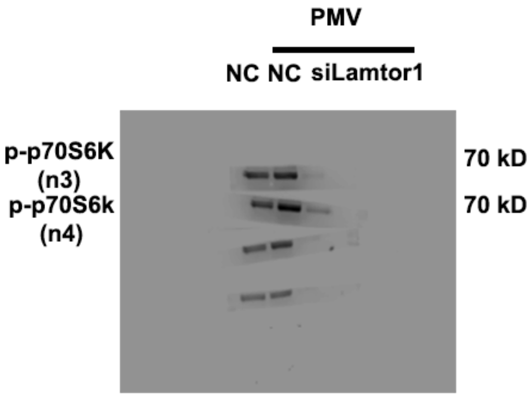

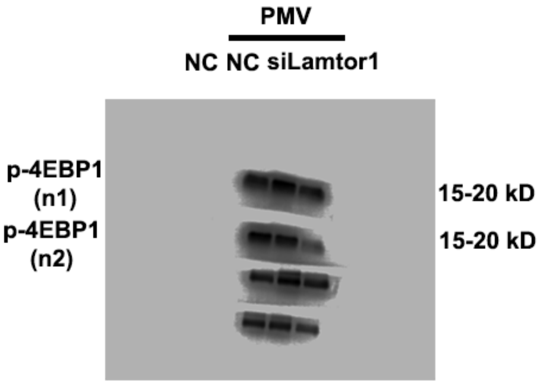

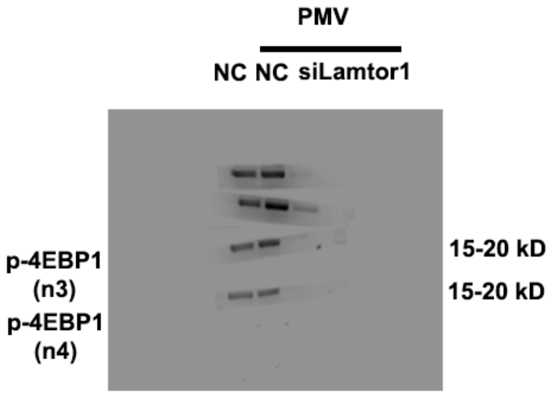

13. Full unedited gel for Figure 2B-GAPDH and Lamtor1.

(1) Full unedited gel for Figure 2B-GAPDH and Lamtor1 presented in the manuscript.

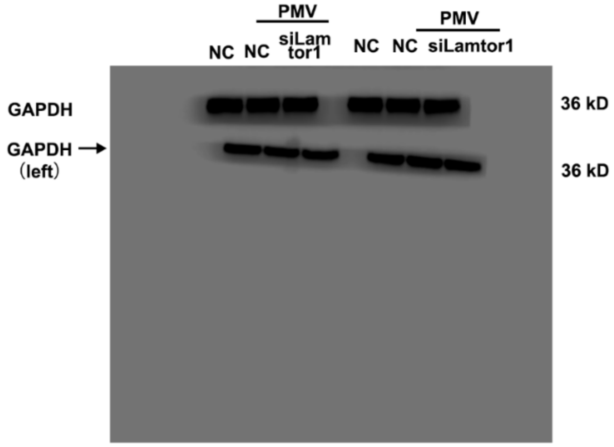

(2) Full unedited gel of Figure 2B- GAPDH and Lamtor1 for all replication.

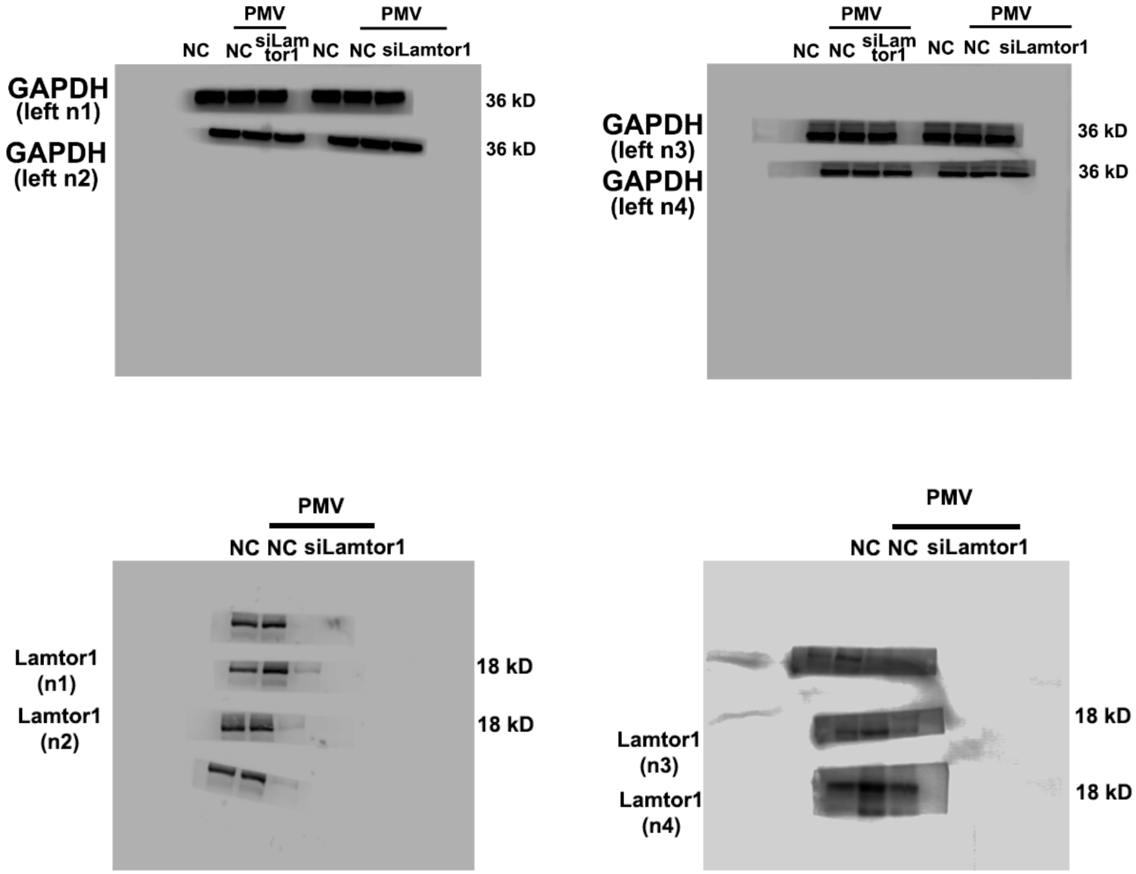

14. Full unedited gel for Figure 2C-SMA.

(1) Full unedited gel for Figure 2C-SMA presented in the manuscript.

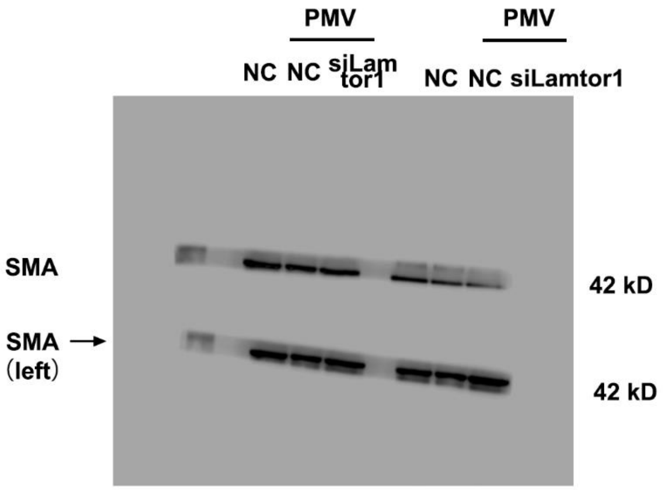

(2) Full unedited gel of Figure 2C-SMA for all replication.

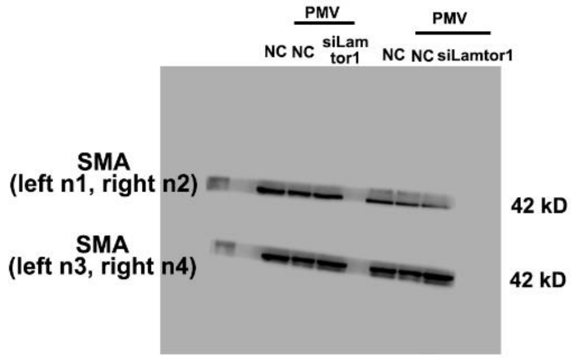

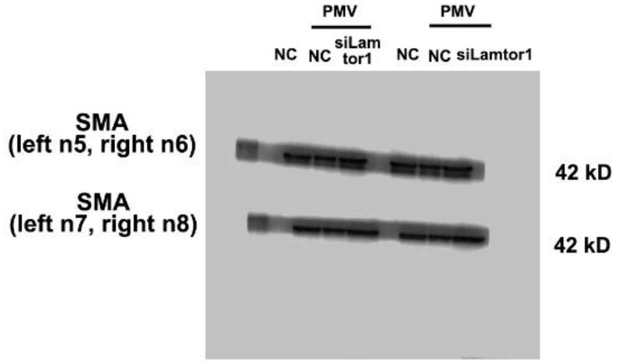

15. Full unedited gel for Figure 2C-Calponin.

(1) Full unedited gel for Figure 2C-Calponin presented in the manuscript.

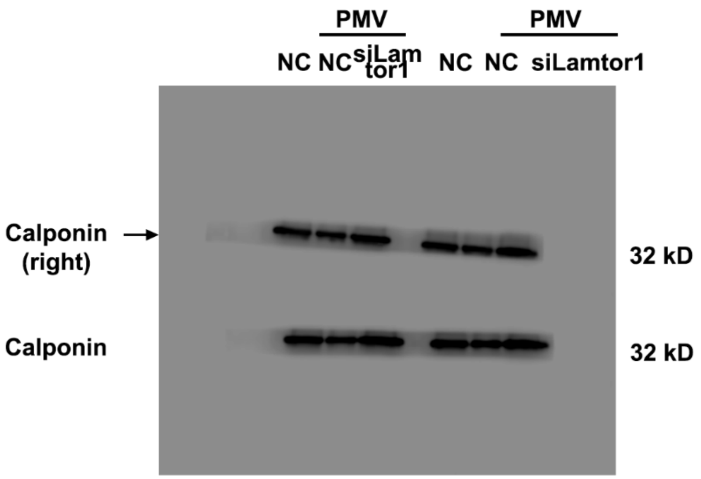

(2) Full unedited gel of Figure 2C- Calponin for all replication.

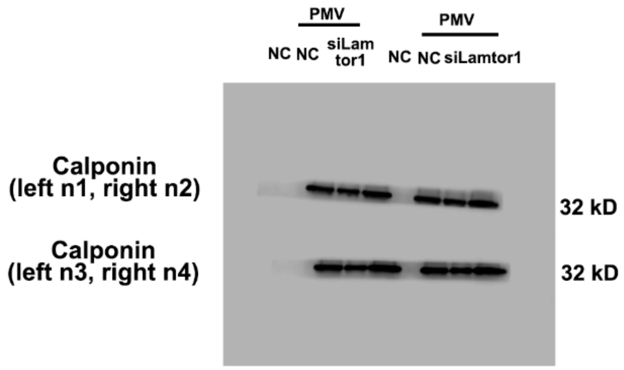

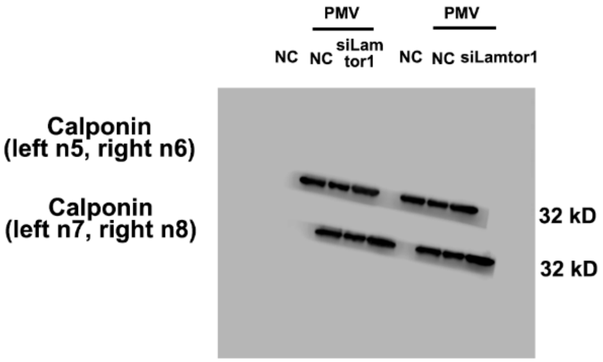

16. Full unedited gel for Figure 2C-SM22.

(1) Full unedited gel for Figure 2C-SM22 presented in the manuscript.

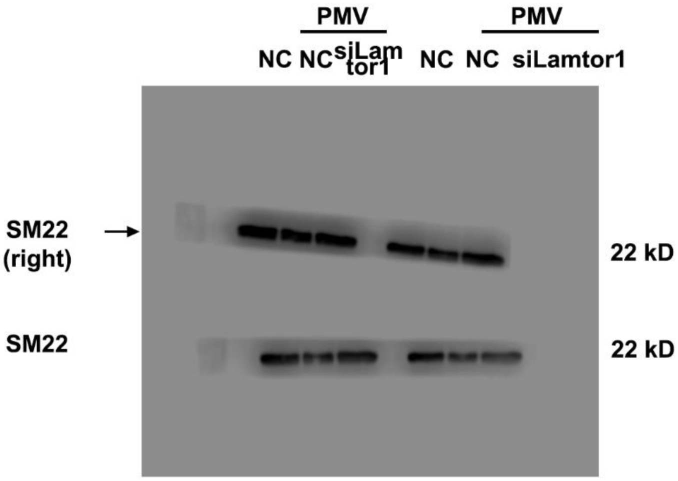

(2) Full unedited gel of Figure 2C- SM22 for all replication.

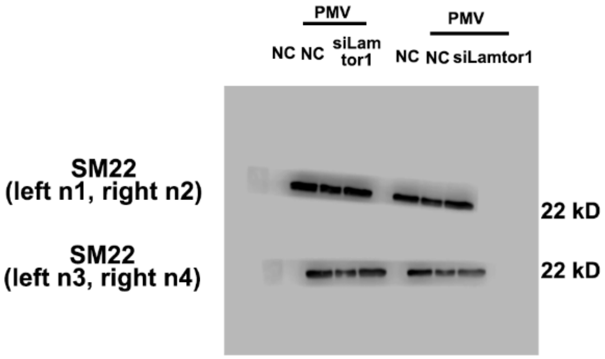

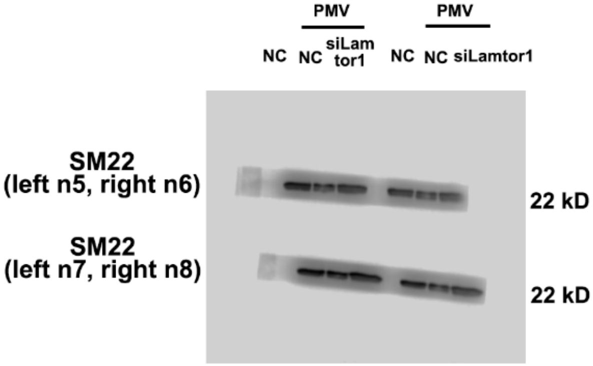

17. Full unedited gel for Figure 2C-GAPDH.

(1) Full unedited gel for Figure 2C-GAPDH presented in the manuscript.

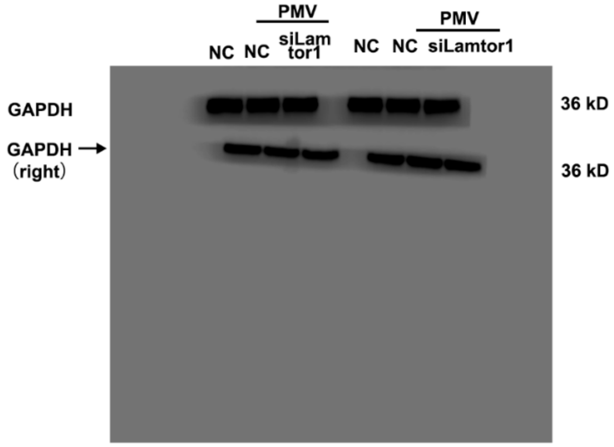

(2) Full unedited gel of Figure 2C- GAPDH for all replication.

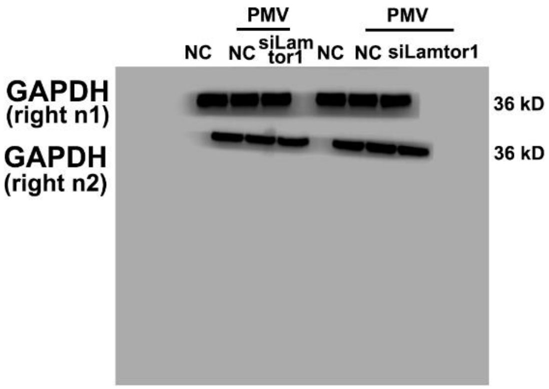

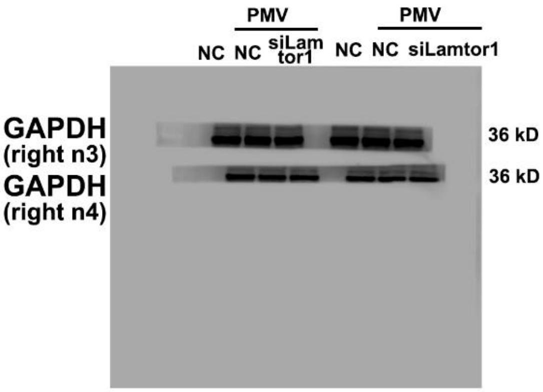

18. Full unedited gel for Figure 3B-p-Src.

(1) Full unedited gel for Figure 3B-p-Src presented in the manuscript.

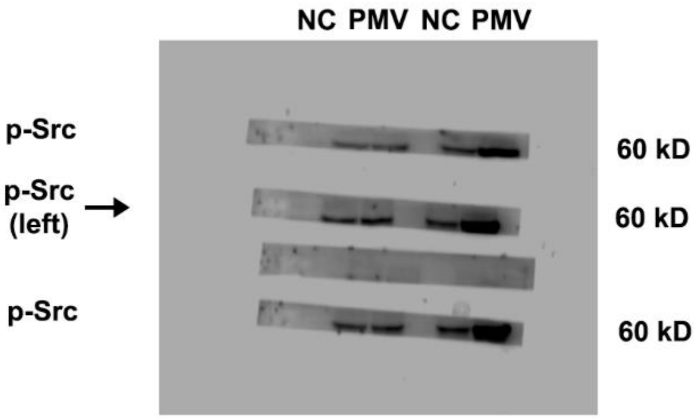

(2) Full unedited gel of Figure 3B-p-Src for all replication.

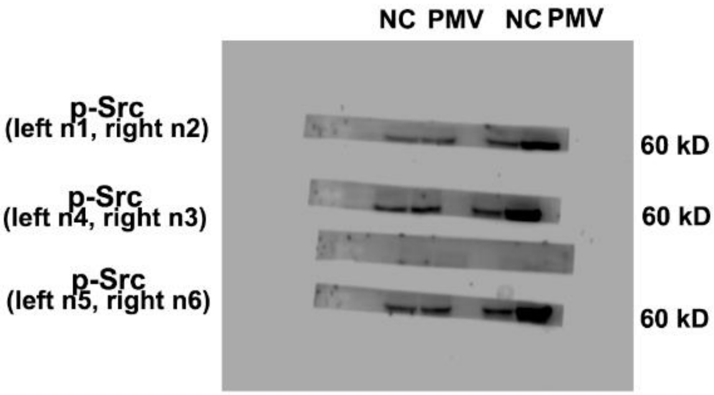

19. Full unedited gel for Figure 3B-Src.

(1) Full unedited gel for Figure 3B-Src presented in the manuscript.

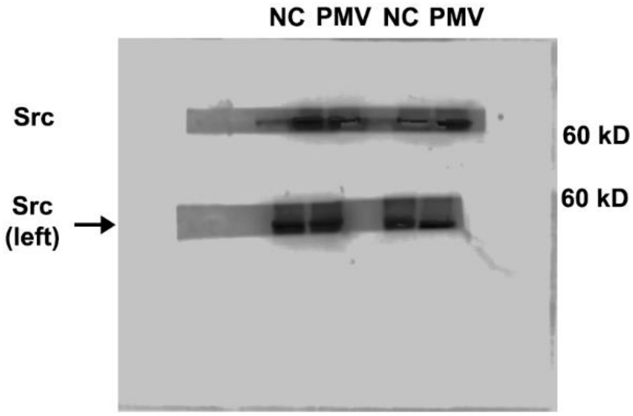

(2) Full unedited gel of Figure 3B-Src for all replication.

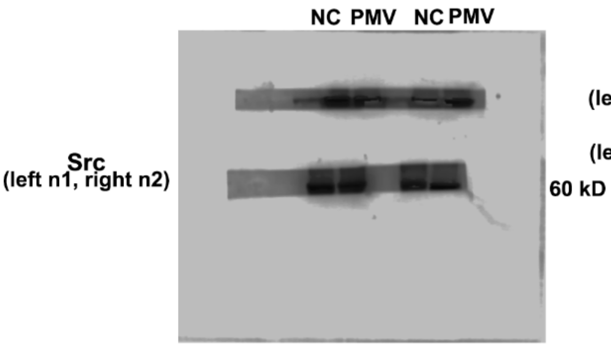

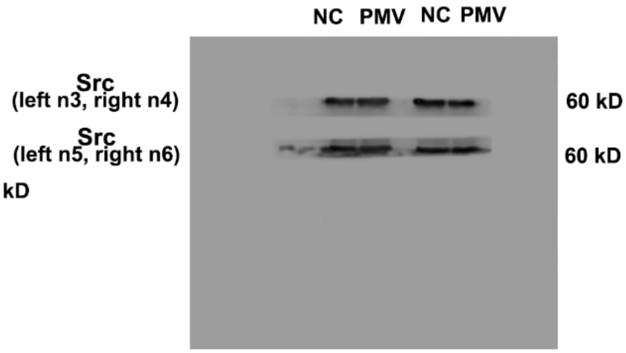

20. Full unedited gel for Figure 3B-GAPDH.

(1) Full unedited gel for Figure 3B-GAPDH presented in the manuscript.

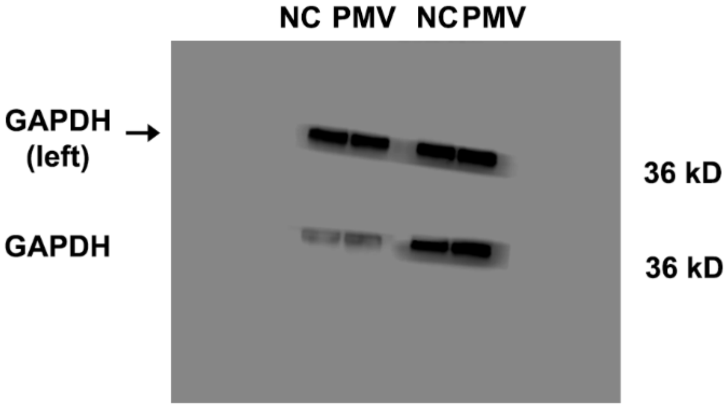

(2) Full unedited gel of Figure 3B- GAPDH for all replication.

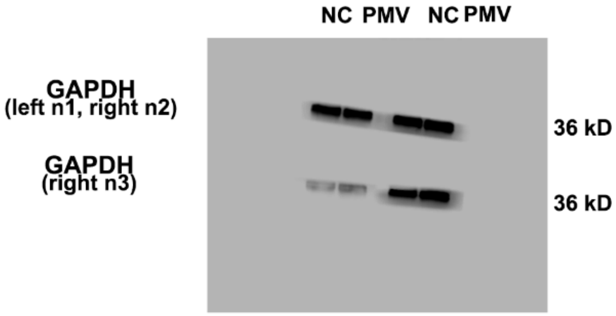

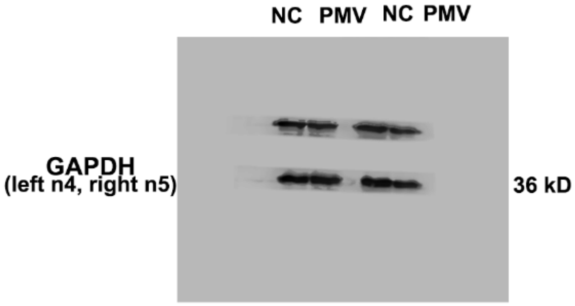

21. Full unedited gel for Figure 4A-p-Src.

(1) Full unedited gel for Figure 4A-p-Src presented in the manuscript.

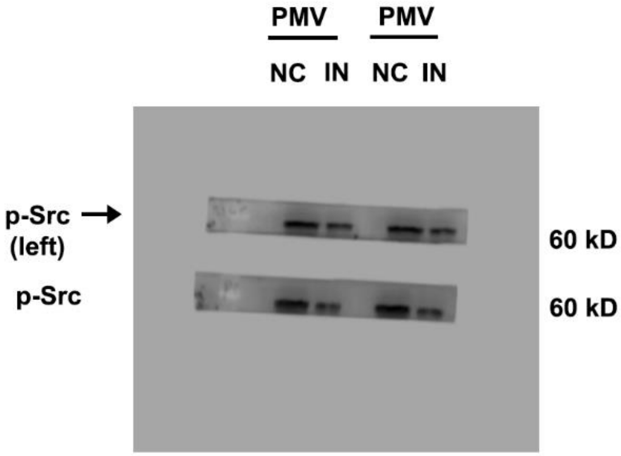

(2) Full unedited gel of Figure 4A-p-Src for all replication.

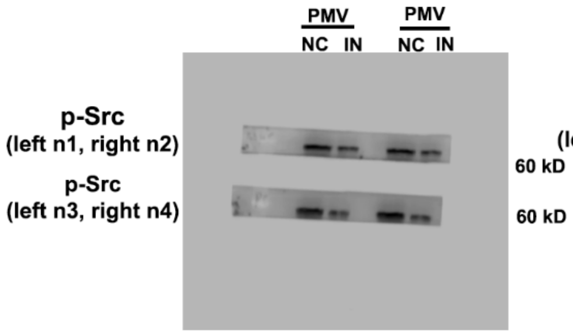

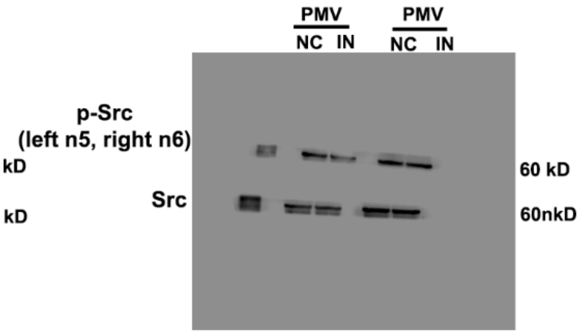

22. Full unedited gel for Figure 4A-Src.

(1) Full unedited gel for Figure 4A-Src presented in the manuscript.

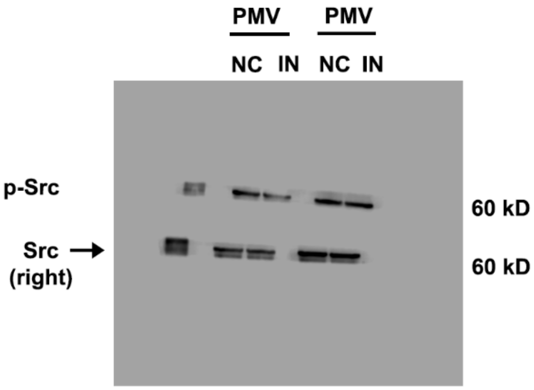

(2) Full unedited gel of Figure 4A-Src for all replication.

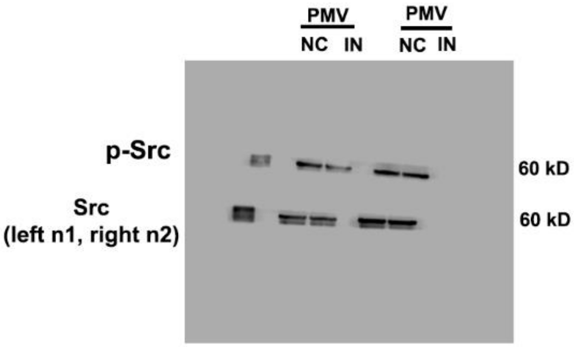

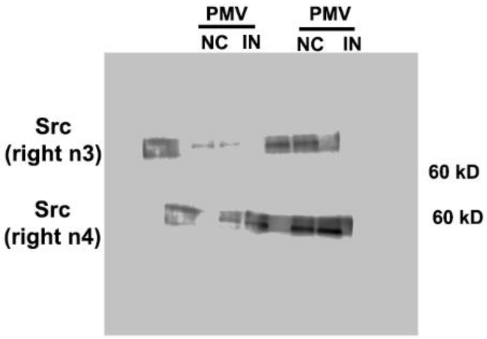

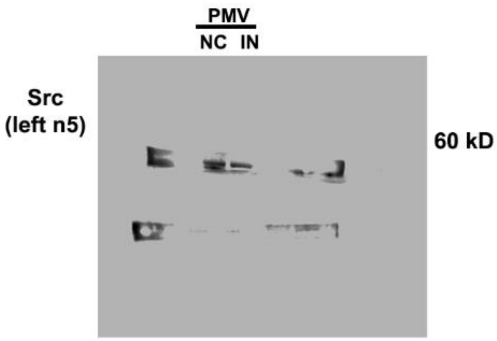

23. Full unedited gel for Figure 4A-GAPDH.

(1) Full unedited gel for Figure 4A-GAPDH presented in the manuscript.

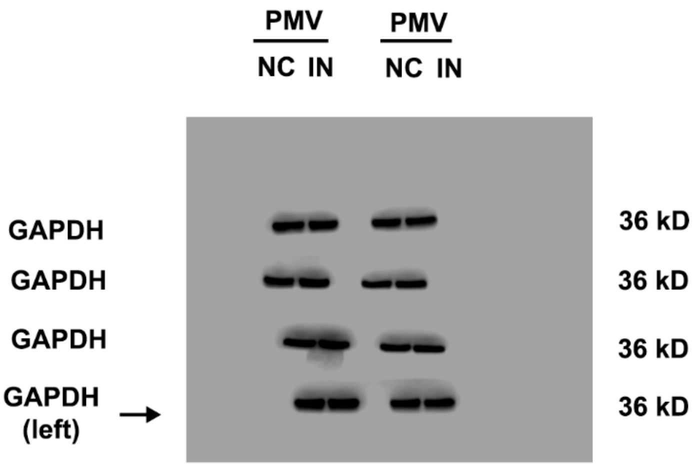

(2) Full unedited gel of Figure 4A- GAPDH for all replication.

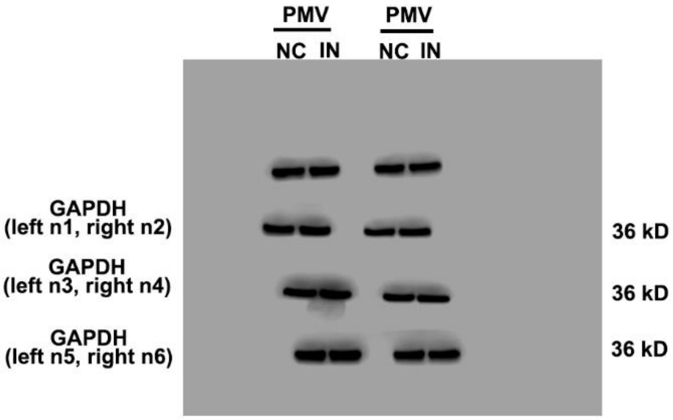

24. Full unedited gel for Figure 4C-Lamtor1.

(1) Full unedited gel for Figure 4C-Lamtor1 presented in the manuscript.

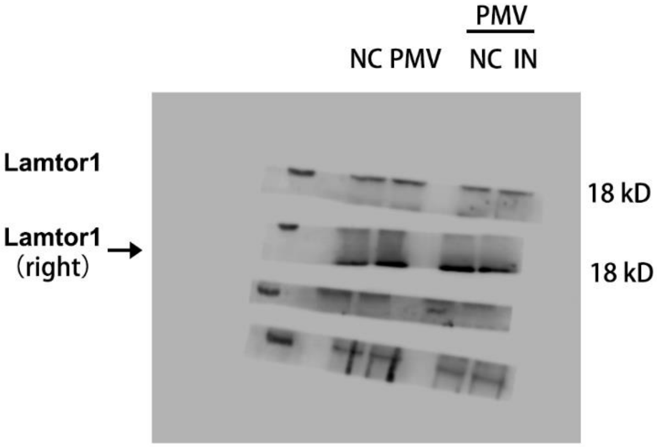

(2) Full unedited gel of Figure 4C-Lamtor1 for all replication.

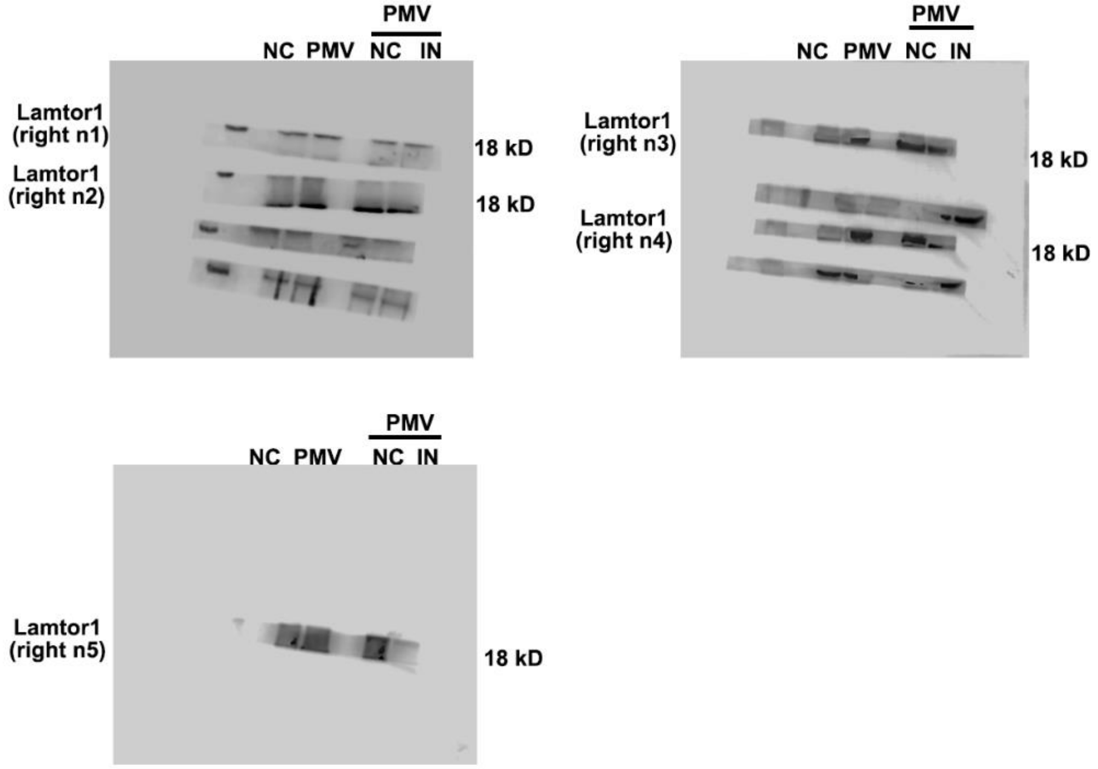

25. Full unedited gel for Figure 4C-GAPDH.

(1) Full unedited gel for Figure 4C-GAPDH presented in the manuscript.

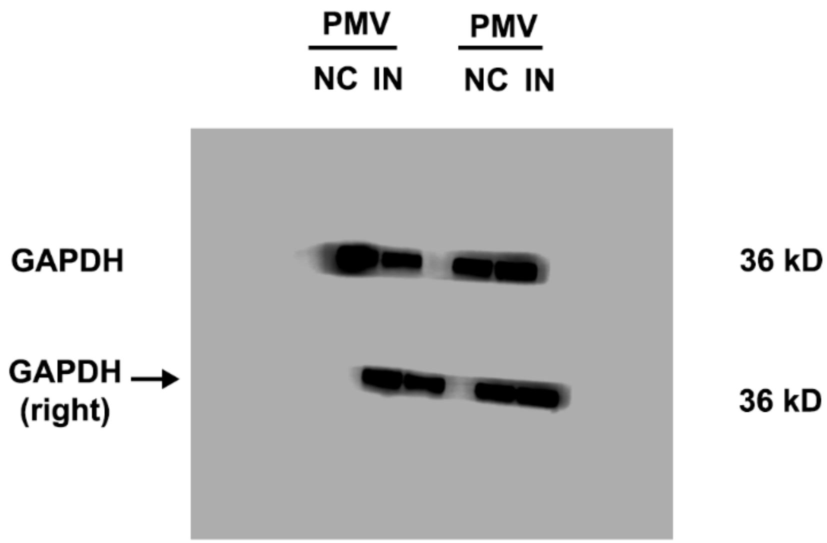

(2) Full unedited gel of Figure 4C- GAPDH for all replication.

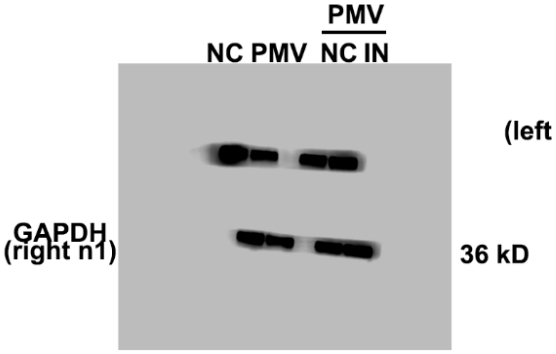

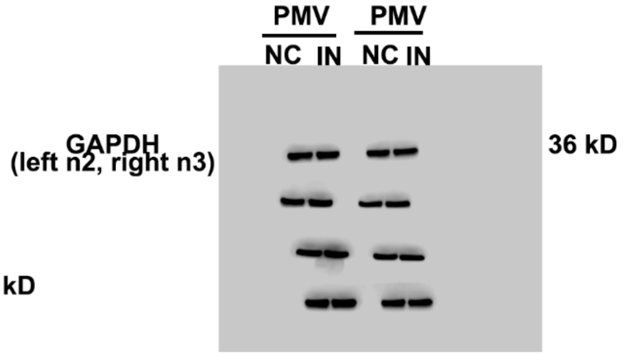

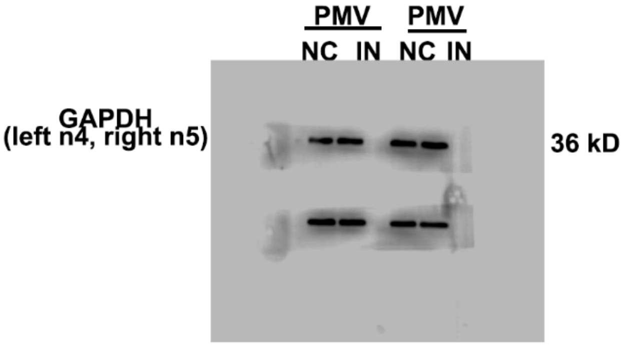

26. Full unedited gel for Figure 4D-mTORC1.

(1) Full unedited gel for Figure 4D-mTORC1 presented in the manuscript

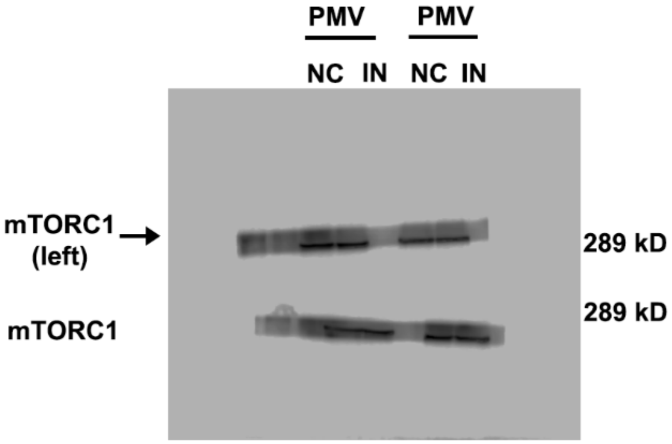

(2) Full unedited gel of Figure 4D-mTORC1 for all replication.

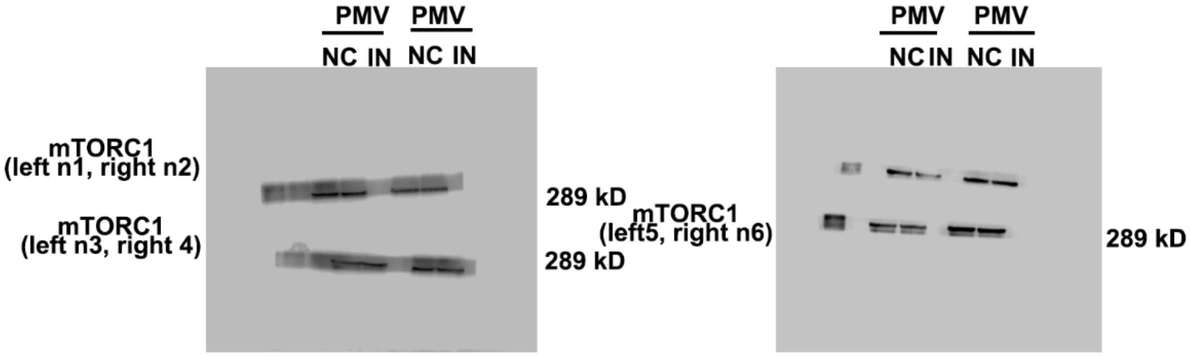

27. Full unedited gel for Figure 4D-p-mTORC1.

(1) Full unedited gel for Figure 4D-p-mTORC1 presented in the manuscript.

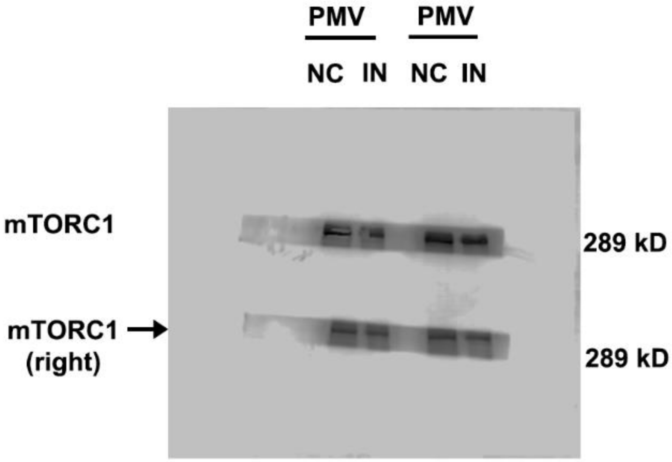

(2) Full unedited gel of Figure 4D-p-mTORC1 for all replication.

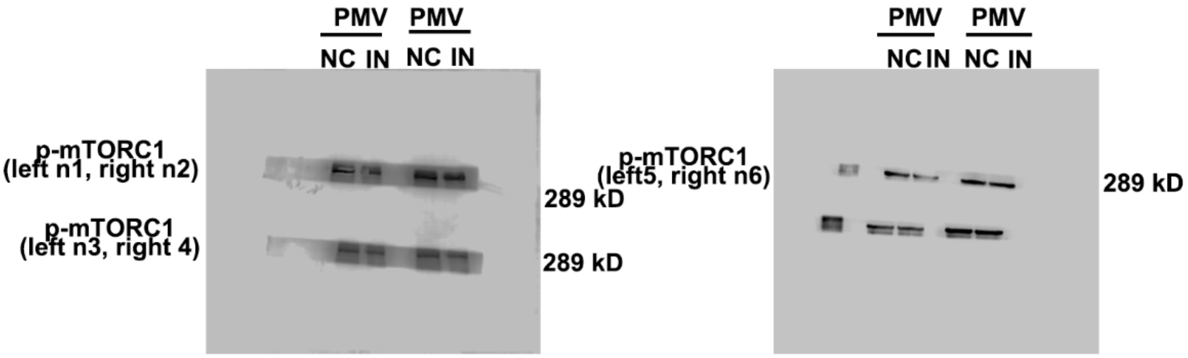

28. Full unedited gel for Figure 4D-p-p70S6K.

(1) Full unedited gel for Figure 4D-p-p70S6K presented in the manuscript.

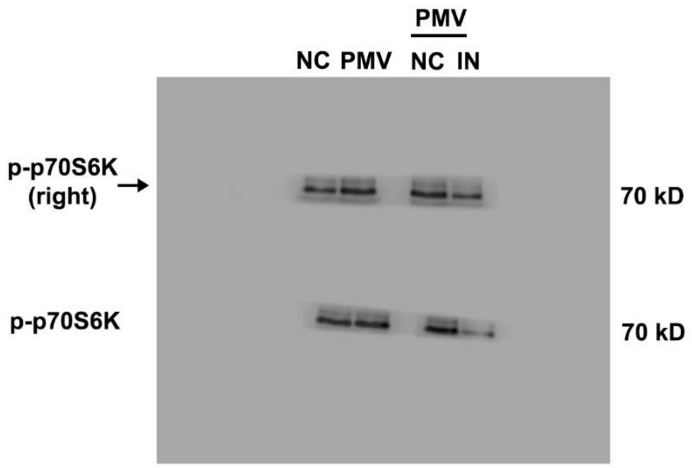

(2) Full unedited gel of Figure 4D- p-p70S6K for all replication.

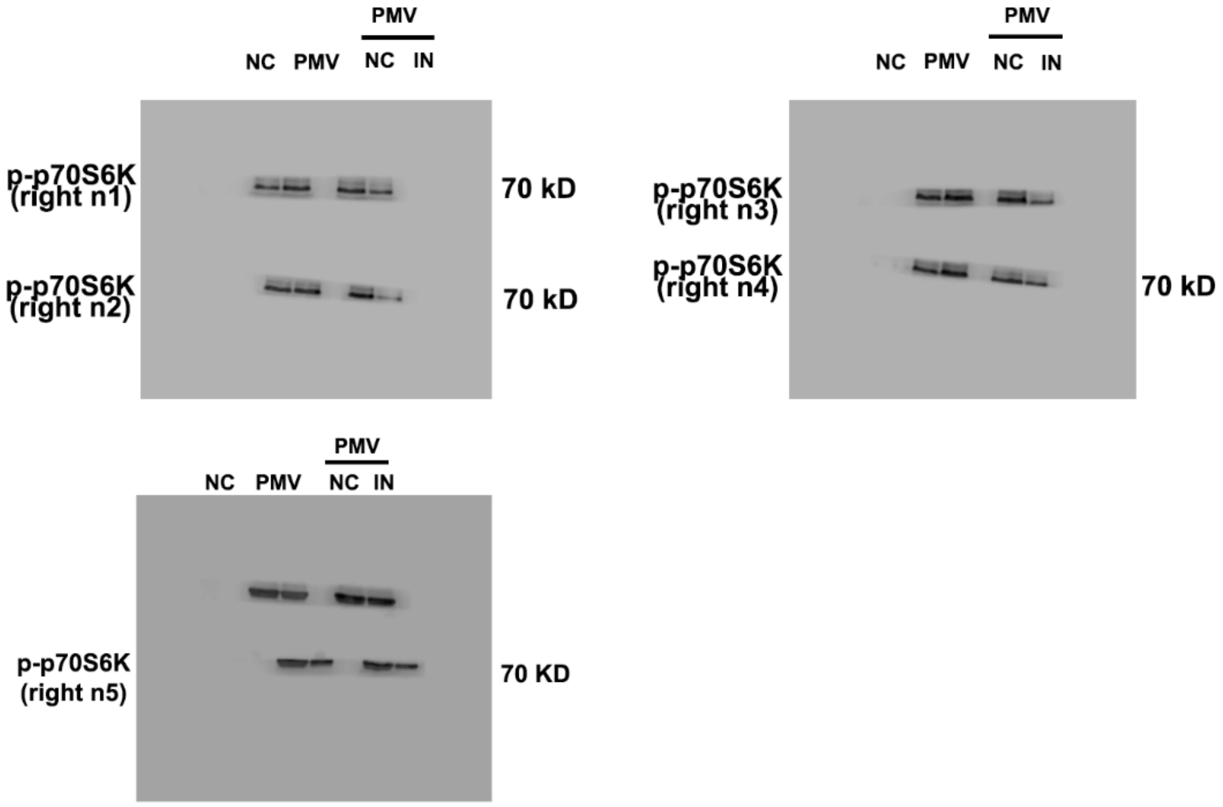

29. Full unedited gel for Figure 4D-p-4EBP1.

(1) Full unedited gel for Figure 4D-p-4EBP1 presented in the manuscript.

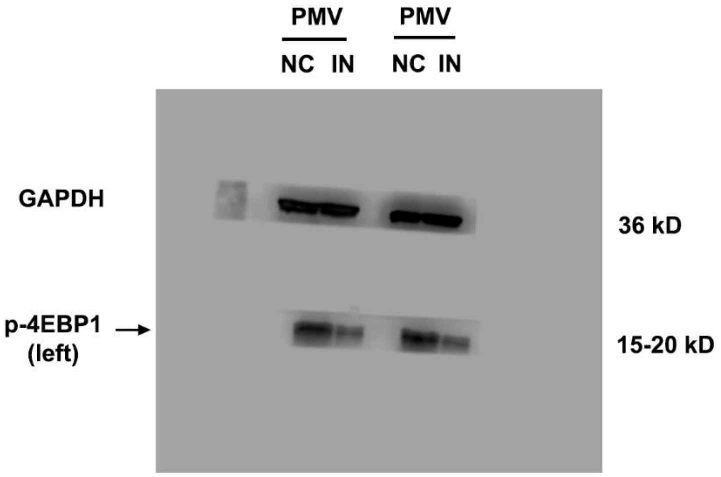

(2) Full unedited gel of Figure 4D- p-4EBP1 for all replication.

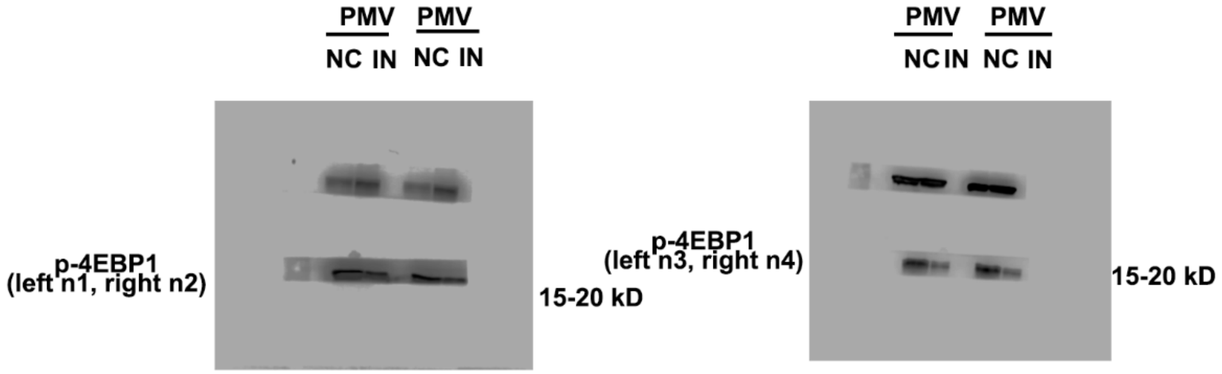

30. Full unedited gel for Figure 4D-GAPDH.

(1) Full unedited gel for Figure 4D-GAPDH presented in the manuscript.

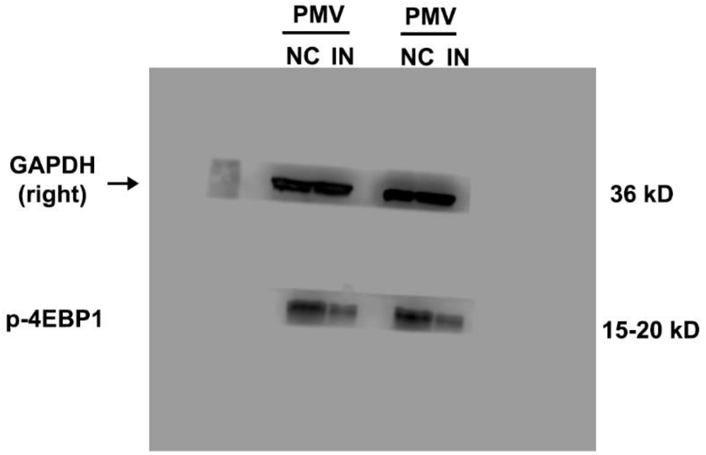

(2) Full unedited gel of Figure 4D-GAPDH for all replication.

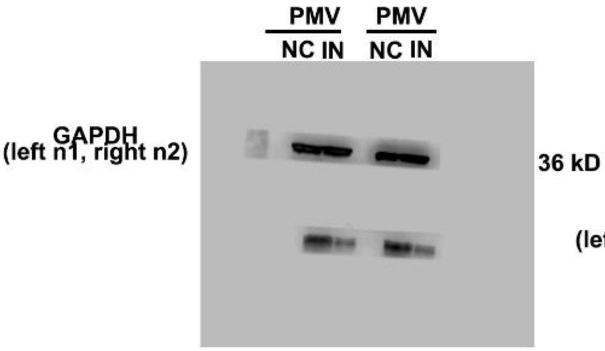

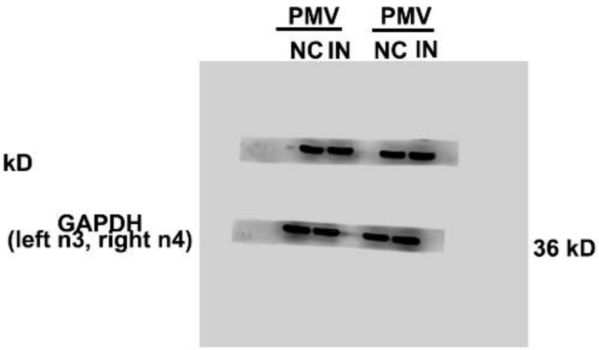

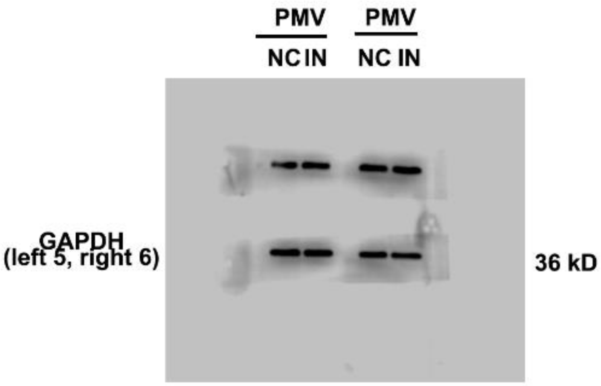

31. Full unedited gel for Figure 4E-SMA.

(1) Full unedited gel for Figure 4E-SMA presented in the manuscript.

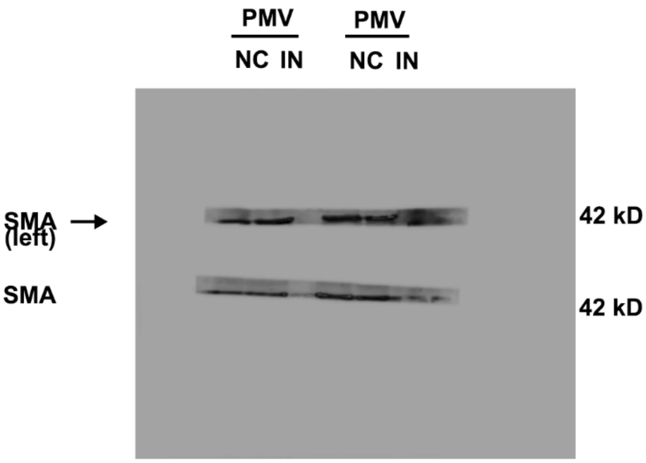

(2) Full unedited gel of Figure 4E-SMA for all replication.

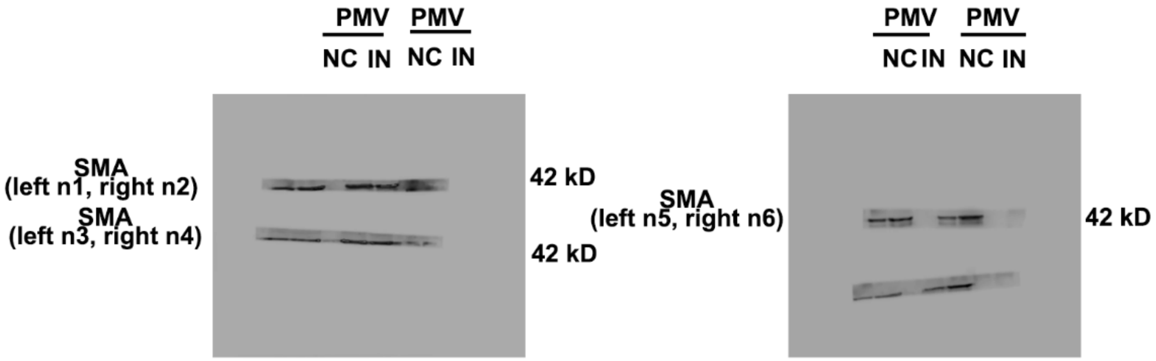

32. Full unedited gel for Figure 4E-Calponin.

(1) Full unedited gel for Figure 4E-Calponin presented in the manuscript.

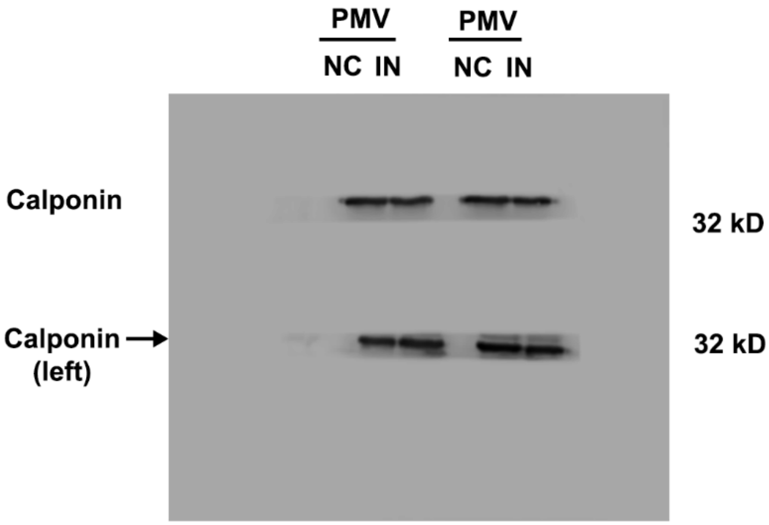

(2) Full unedited gel of Figure 4E- Calponin for all replication.

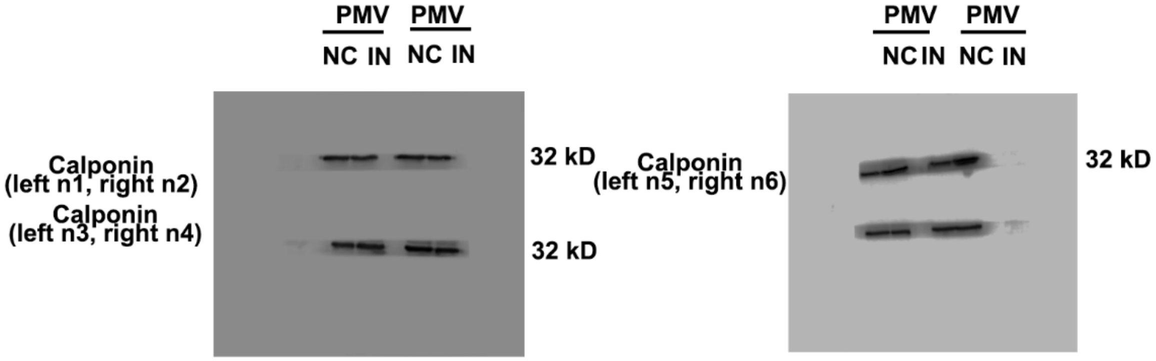

33. Full unedited gel for Figure 4E-SM22.

(1) Full unedited gel for Figure 4E-SM22 presented in the manuscript.

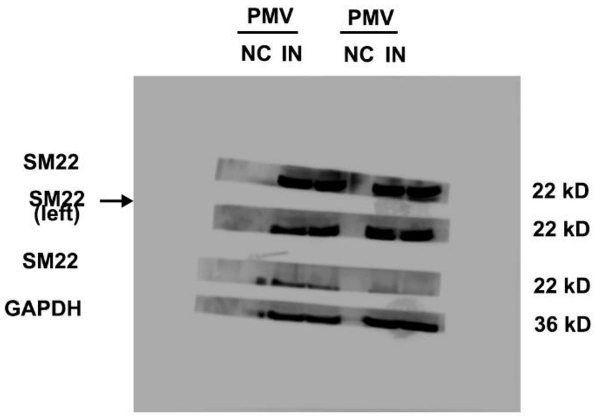

(2) Full unedited gel of Figure 4E- SM22 for all replication.

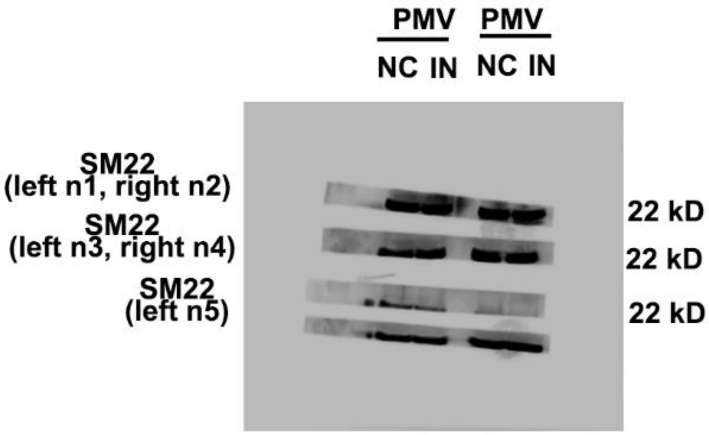

34. Full unedited gel for Figure 4E-GAPDH.

(1) Full unedited gel for Figure 4E-GAPDH presented in the manuscript.

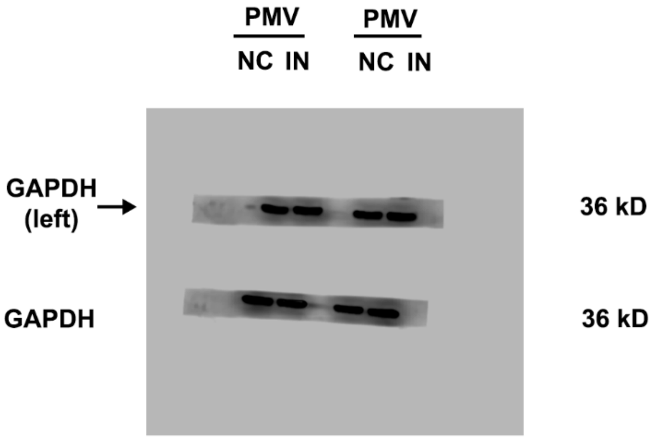

(2) Full unedited gel of Figure 4E- GAPDH for all replication.

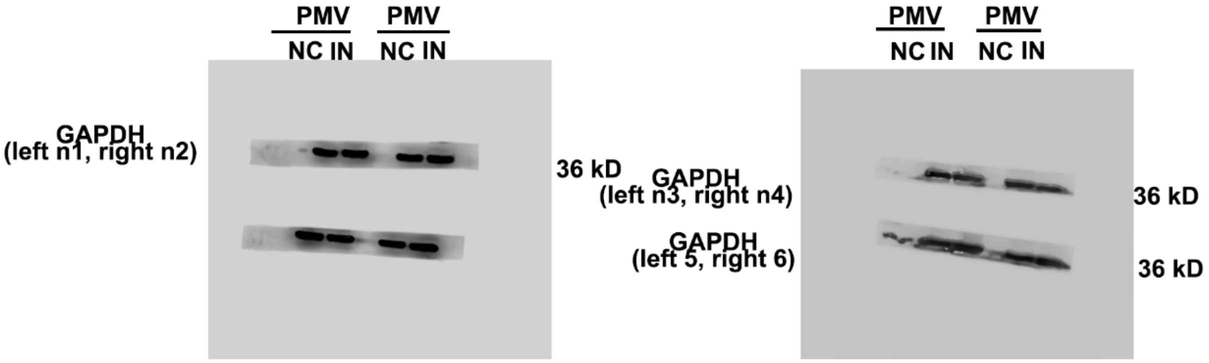

Supplement: Supplementary file 2 [file Image_1.PDF]
